# Psychophysiological effects of low-frequency sound mediated by room size, room shape, and stimulus duration

**DOI:** 10.3389/fcogn.2026.1806052

**Published:** 2026-05-20

**Authors:** Paul Oomen, Rona Geffen, Daniela Gentile, Nour Atassi, Bashar Farran, Christoph Braun, Veronica Cuevas Villanueva, Luka Nadiradze, Máté Csanád, Amira Val Baker

**Affiliations:** 1 The Works Research Institute, Amsterdam, Netherlands; 2 Adult MEG Laboratory, MEG-Center, Faculty of Medicine, University of Tübingen, Tübingen, Germany; 3 Department of Atomic Physics, Eötvös Loránd University, Budapest, Hungary

**Keywords:** autonomic nervous system, electroencephalography, electromyography, emotional response, experimental acoustics, galvanic skin response, heart rate variability, spatial audio

## Abstract

Music and sounds elicit a wide range of emotions and activate numerous psychological and physiological effects associated with the functioning of the autonomic nervous system (ANS) and involved in the maintenance of homeostasis. As such, sound interventions can play an important role in supporting human wellbeing and improve outcomes in medical patients. Psychophysiological responses are dependent on the stimulus type, frequency, duration, and spatial conditions of the acoustic environment. To arrive at a more articulate understanding of these dependencies, the effects of singing bowl sounds at 40 Hz, 73 Hz, and 110 Hz were investigated by monitoring physiological, behavioral and emotional responses in healthy adult subjects (*n* = 59). Singing bowl sounds were spatially projected in three virtual room sizes (small, medium, large) and eight virtual room shapes (pyramid, tetrahedron, cube, octahedron, dodecahedron, icosahedron, cuboctahedron, and sphere). Overall, exposure to low-frequency singing bowl sounds resulted in a significant increase in positive emotions and a significant decrease in negative emotions across all conditions. Contrary to other studies, we found no discrete effects related to the stimulus type or frequency. Significant differences in electroencephalography (EEG), heart rate variability (HRV) and electromyography (EMG) were consistently dependent on a combination of stimulus frequency and spatial condition, i.e. the resonance of a sound in a room with a particular size and shape. Significant differences in localized EEG power were strongly correlated to localized amplitude deviations of sound waves, while the phase shift of sound wave frequency was predictive of EEG frequency. Significant differences in HR and EMG were strongly correlated to the mean, variability and standard deviation of phase distortions of sound waves. Discrete effects of room shape were observed in galvanic skin response (GSR). Arousal was significantly decreased in a cube and cubeoctahedron, regardless of the frequency. Discrete effects of stimulus duration were observed in GSR and EMG. Arousal was significantly increased during the first 15 min of exposure and significantly decreased until 40 min, regardless of the frequency and spatial condition. We discuss the implications of these findings for future research and therapeutic practices.

## 1 Introduction

Sounds and music elicit a wide range of emotions (Lundqvist et al., 2008; Thompson et al., 2016) and activate numerous psychological and physiological effects (Koelsch et al., 2008, 2016; Kulinski et al., 2022; Mir et al., 2021; Stewart et al., 2019; Valenti et al., 2012), associated with the functioning of the autonomic nervous system (ANS) and involved in the maintenance of homeostasis (Juslin and Västfjäll, 2008; Reybrouck et al., 2021). Auditory stimulation acts on the cerebral cortex and limbic system through the hypothalamic pathway (Blood and Zatorre, 2001; Gordan et al., 2015). Emotional response to auditory stimuli is mediated by the connectivity between the hypothalamus and the hippocampus (Hou et al., 2017), inducing various endocrine responses and regulating the release of neurotransmitters (Akimoto et al., 2018; Chanda and Levitin, 2013; Ooishi et al., 2017; de Witte et al., 2020). As such, sound interventions can play an important role in supporting human wellbeing and improve outcomes in medical patients by reducing pain perception, lowering anxiety and stress levels, and stabilizing physiological parameters (Cecyli et al., 2024; Lorek et al., 2023). The ability of sounds and music to modify the activity in the hippocampus (Koelsch, 2014) gives rise to methods for treating mental disorders, such as depression (Fachner et al., 2012; Torun et al., 2020), anxiety (Bradt et al., 2013; Karakul and Bolişik, 2018), post-traumatic stress disorder (Landis-Shack et al., 2017) and personality disorder (Haslam et al., 2022), and inducing functional changes in neurodegenerative conditions, such as Alzheimer's and Parkinson's diseases (Bleibel et al., 2023; Machado Sotomayor et al., 2021; Matziorinis and Koelsch, 2022).

Sound interventions are categorized as sound therapy, music therapy or vibroacoustic therapy (VAT), primarily depending on the type of stimulus and delivery method (Saskovets et al., 2025; Vincent et al., 2025). VAT uses low-frequency sound vibrations between 30–120 Hz for promoting physical and emotional wellbeing (Punkanen and Ala-Ruona, 2012), addressed through tactile transduction and/or sympathetic resonance of the body with the incoming low-frequency waves. Emerging applications of VAT include its utilization in treatment of depression (Sigurdardóttir et al., 2019), coronary heart disease (Du et al., 2025), inflammatory diseases (Ahn et al., 2024), respiration failure (Konkayev and Bekniyazova, 2023) and emotion regulation in autistic children (Moore et al., 2025). An important aspect of both music and vibroacoustic therapy is the active therapeutic relationship, as distinct from sound therapy which has been considered a passive experience where the therapeutic effect relies on specific tones, frequencies and vibrations (Chockboondee et al., 2024; Imbriani, 2017; Mingorance et al., 2021). Singing bowls are considered an effective stimulus for sound therapy, and were found to decrease tension, anger, fatigue and depressed mood (Goldsby et al., 2016), increase relaxation and positive mood (Kim and Choi, 2023) and improve brain coherence and autonomic balance (Bidin et al., 2016; Sundharakumar et al., 2021; Trivedi et al., 2019).

Common measures to assess psychophysiological responses to sound stimuli include electroencephalography (EEG), heart rate (HR), heart rate variability (HRV), electromyography (EMG) and galvanic skin response (GSR). These methods have been used to infer emotional responses along the dimensions of arousal and valence. EEG records the spontaneous electrical activity of the brain and is used to assess different aspects of cortical auditory processing (Biasiucci et al., 2019). The frequency of brain activity is indicative of mental state and cognitive load, where delta (<4 Hz) is associated with sleep and dream states; theta (4–8 Hz) with drowsiness, deep relaxation and meditation; alpha (8–12 Hz) with reflective and restful states, where alpha power and brain activity are inversely related; beta (13–30 Hz) with increased alertness, cognitive processes and active states; and gamma (>30 Hz) with problem solving, working memory and higher levels of attention and concentration (Kučikiene and Praninskiene, 2018; Nayak and Anilkumar, 2024; Woaswi et al., 2016). EEG can reflect both arousal and valence, where arousal is linked to overall activation and valence can be inferred from topographical analysis, and has been associated with frontal asymmetry (Coan and Allen, 2004). However, there is high sensitivity to individual differences between subjects which complicates generalizing the interpretation of EEG signals (Valderrama and Sheoran, 2025). Beat frequencies of singing bowls, i.e., rhythmic pulsations created by the interference of different sound waves produced by the bowls, were found to promote an increase in theta activity and synchronization, effectively facilitating meditation and relaxation (Ahn et al., 2019; Kim and Choi, 2023). In response to singing bowl interventions, some studies observed increased high-alpha and low-beta activity corresponding to awakening and relaxation (Kim and Choi, 2023; Sundharakumar et al., 2021), while others reported a significant decrease in alpha, beta and gamma activity and associated positive effects on emotional valence (Walter and Hinterberger, 2022). A lack of standardized protocols for emotion elicitation and classification of EEG has been observed, which prevents effective cross-study comparisons (Gkintoni et al., 2025).

HRV consists of variations in the time intervals between consecutive heartbeats, referred to as the RR interval. HRV is related to HR in a cycle-length dependence, i.e., when HR increases the time between heartbeats is less and HRV decreases (McCraty and Shaffer, 2015; Shaffer and Ginsberg, 2017). HRV is defined as multiple parameters across the time domain, e.g., standard deviation of the normal-to-normal range (SDNN) and root mean square of successive RR interval differences (RMSSD); frequency domain, e.g., low frequency/high frequency (LF/HF) ratios; and nonlinear indices, e.g., Poincare plots, each reflecting different aspects of autonomic balance (Shaffer and Ginsberg, 2017). HRV is considered an indicator of homeostasis (Arakaki et al., 2023; Gordan et al., 2015; Gullett et al., 2023) and has been used to assess emotional valence and stress levels in subjects (Attar et al., 2021; Choi et al., 2017; Kim et al., 2018), where lower HRV indicates higher stress and arousal and higher HRV indicates increased relaxation. Singing bowl interventions provided better depth of relaxation than supine silence and a significant decrease in stress levels, as indicated by a decrease in HR and increase in HRV (Trivedi et al., 2019). However, the field is methodologically fragmented with no consensus on the most effective short-term (<5 min) indices for tracking rapid arousal shifts (Zimatore et al., 2026).

EMG measures muscle activity of subjects (Okamoto and Murao, 2023), where increased relaxation was associated with decreased muscle tension (van Criekinge et al., 2021; Scartelli, 1984). Music listening and rhythmic auditory stimulation were able to activate various motor-cognitive functions as well as modulate neural plasticity (Park et al., 2023; Ulloa, 2022) and accelerate the recovery process of fatigued muscles (van Criekinge et al., 2021). While facial EMG is typically considered more sensitive to affective valence, EMG activity in skeletal muscles more broadly reflects changes in general physiological activation and tension associated with emotional states (Cacioppo et al., 2007). Therefore, arm EMG can be interpreted as a measure of overall arousal rather than specific emotional valence.

GSR measures the electrical conductivity of the skin or electro-dermal activity (Boucsein, 2012; Markiewicz et al., 2022). Psychophysiological stress and emotional responses activate sweat glands, thereby increasing perspiration and skin conductivity (Greco et al., 2017). The signal can change quickly (phasic), such as in response to stimulation, or slowly (tonic), such as in adaptation to the environment or to a general stress level (Boucsein, 2012). GSR is considered a reliable indicator of autonomic expressions of emotion in the auditory domain, showing increased electrodermal activity in emotional changes elicited by pleasant or unpleasant sound stimuli (Bradley and Lang, 2000), emotionally intense music (Sudheesh and Joseph, 2000) unfamiliar musical stimuli (Kuan et al., 2018) and unexpected musical chords eliciting excitement and surprise (Koelsch et al., 2008). Singing bowl sounds induced a significant decrease in the level of both phasic and tonic GSR, indicating a lower level of arousal and a reduction in anxiety and involuntary mental activity (Bidin et al., 2016). GSR measures arousal and not valence, as it does not distinguish between positive and negative arousal. It is also sensitive to non-emotional factors like temperature and physical movement (Baig and Kavakli, 2019; Kołodziej et al., 2019). It has been noted that many studies record complex GSR features but fail to fully actualize emotion recognition, often relying on a single mean value (Kołodziej et al., 2019). Using a combination of EEG, HRV, EMG, and GSR provides a more robust and multi-faceted approach to assessing both intensity and direction of emotional states, typically achieving classification accuracies above 70% and sometimes exceeding 90% when combined (Baig and Kavakli, 2019).

Another fundamental aspect of sound interventions is the study of sound frequencies and its embedding in music and sounds to affect mental and physiological health (Bartel and Mosabbir, 2021). While deeply connected with the broader study of sound vibration, tempo and amplitude, various discrete effects have been associated with stimulation at specific frequencies. Notably, 40 Hz has been shown to mediate thalamo-cortical interactions (Llinas and Ribady, 1993; Naghdi et al., 2015), increase gamma oscillations in the frontal, temporal and central regions (Jirakittayakorn and Wongsawat, 2017) and activate mechanisms in the brain involving auditory processing (Parciauskaite et al., 2019; Pastor et al., 2002). 40 Hz stimulation has been used to reduce inflammation, pain and anxiety (McMakin, 2017) and for treatment of Alzheimer's disease (Cimenser et al., 2021; Clements-Cortes et al., 2016; Koike et al., 2004) and Parkinson's disease (Mosabbir et al., 2020). 73 Hz has been reported to stimulate subcortical areas, particularly the hypothalamus (Nogier, 2021), indicating it as a trigger for hormonal balance and emotional reactions. 73 Hz stimulation has been used to treat eating and endocrine disorders, menstrual cycle disorders and menopause-related problems (Nogier, 2014). Other studies investigated effects of 110 Hz on physiological mechanisms and neuronal activity. 110 Hz stimulation induced a significant decrease in activity in the left temporal lobe and an asymmetrical shift to right hemispheric dominance in the prefrontal cortex, associated with emotional processing (Cook et al., 2008), and an increase in cardiac autonomic function (Hori et al., 2005). 110 Hz has been used for sound-induced gene regulation (Kumeta et al., 2018), promotion of wound healing (Katoh, 2023) and cellular homeostasis (Blackman et al., 1985).

It has been shown that other musical parameters, such as changes in tempo, tone and harmony, induce different levels of emotional arousal (Arjmand et al., 2017; Fernández-Sotos et al., 2016; van der Zwaag et al., 2011). Pleasantness of musical stimuli was associated with increased frontal midline theta activity in subjects (Juslin and Västfjäll, 2008; Sammler et al., 2007) and temporal theta oscillations inducing local gamma oscillations in the intracranial regions of the reward circuit (Lv et al., 2024). Prior studies on emotional responses to sound have primarily focused on the relationship to sound parameters such as loudness, frequency and wavetype (Asutay et al., 2012). Listening to static, fixed and meaningless sounds showed that valence was mainly affected by intensity, while arousal was affected by the sharpness of the sound (Västfjäll, 2012). Increase in sound intensity was found to correspond with an increase in hearing-evoked gamma band response (Ott et al., 2013). Variations in intensity of the same frequencies significantly modulated alpha and beta activity in subjects, while variations in the time period modulated the amplitude of theta waves in the frontal, temporal and parietal regions (Di et al., 2018). Sound intensity levels also affected cardiac autonomic regulation differently depending on the type of music stimulus (Do Amaral et al., 2016).

Spatial conditions, such as the room acoustics and the positioning and orientation of sound sources and subjects within the room, are known to strongly affect the properties of sound waves (Barile et al., 2023; Reinten et al., 2017; Toole, 2008), but only few studies have investigated its psychophysiological impact. Spatialization of a 333 Hz stimulus was reported to affect changes in the parietal and occipital lobes and increase alpha activity in the right hemisphere (Qin et al., 2020). Spatial sound projections of tuning fork sounds in geometric shapes (pyramid, cube, and sphere) affected brain topology of subjects, decreased activity and increased connectivity in alpha and beta-1 bands, along with a decrease in blood pressure and heart rate compared to a stereo projection (Geffen and Braun, 2024). Investigation of psychophysiological response to acoustic simulation of different room sizes showed that a small room size evoked higher valence and lower arousal in subjects when listening to natural sounds associated with positive presence, and reached significantly higher values of safety confirmed by physiological markers (Tajadura-Jiménez et al., 2010).

It has been suggested that psychophysiological responses induced by auditory stimuli depend not only on the physical properties of the sound, but also on the listener and the context (Asutay et al., 2012; Juslin and Västfjäll, 2008). Indeed, physiological expression and therapeutic effectiveness were found to be strongly mediated by familiarity and enjoyment of the music by subjects and independent of the music's emotional content (Lv et al., 2024). Furthermore, the sound stimulus is significantly affected by sound wave deviations that are involuntarily caused by the presence of the subject in the acoustic environment, i.e., absorption, diffraction and scattering of the sound waves by the human body, resulting in a nonlinear dynamic interaction of the sound stimulus and the subject. Acoustic deviations in the near-field of the subject amounted to an uncertainty of *u* = 24.64%, while individual traits of the subject explained > 55% of the resulting amplitude deviation and phase distortion (Oomen et al., 2026).

**Figure 1:**
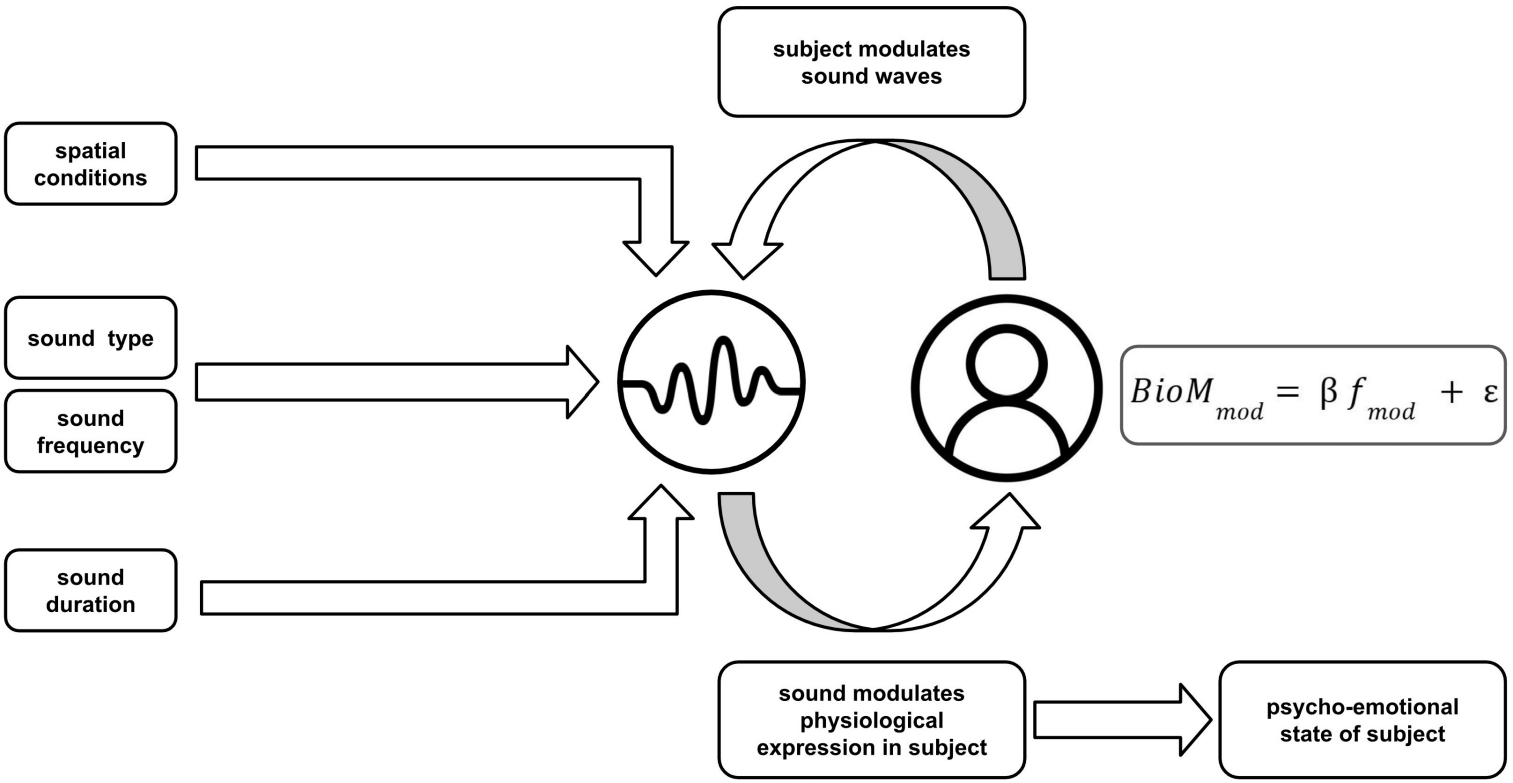
The physioacoustic model: physiological expression in subjects and associated psycho- emotional states are dependent on the type, frequency, intensity, spatial conditions and duration of the sound stimulus. The transfer function from a sound stimulus at a given frequency *f* to a change in physiological expression *BioM* is interdependent on deviations in the sound field that are involuntarily caused by the presence of the subject. The presence of the subject in the acoustic environment is shown to recursively interfere with the sound stimulus, establishing a non-linear dynamic interaction.

Psychophysiological responses to sound are thus dependent on the stimulus type, frequency and intensity, and various spatial conditions including the presence of the subject in the acoustic environment. However, to what extent and in which way these dependencies contribute to outcomes in sound interventions has not been specifically investigated in prior studies. For the purpose of this study, we introduce the *physioacoustic* model, referring to the interaction of acoustic phenomena and physiological expressions as shown in Figure 1. On the basis of the physioacoustic model, we investigated responses in healthy adult subjects according to four aspects of the sound stimulus: i) frequency; ii) room size; iii) room shape; and iv) duration. Furthermore, physiological changes across the investigated conditions were correlated to acoustic deviations to determine their interdependence.

**Figure 2:**
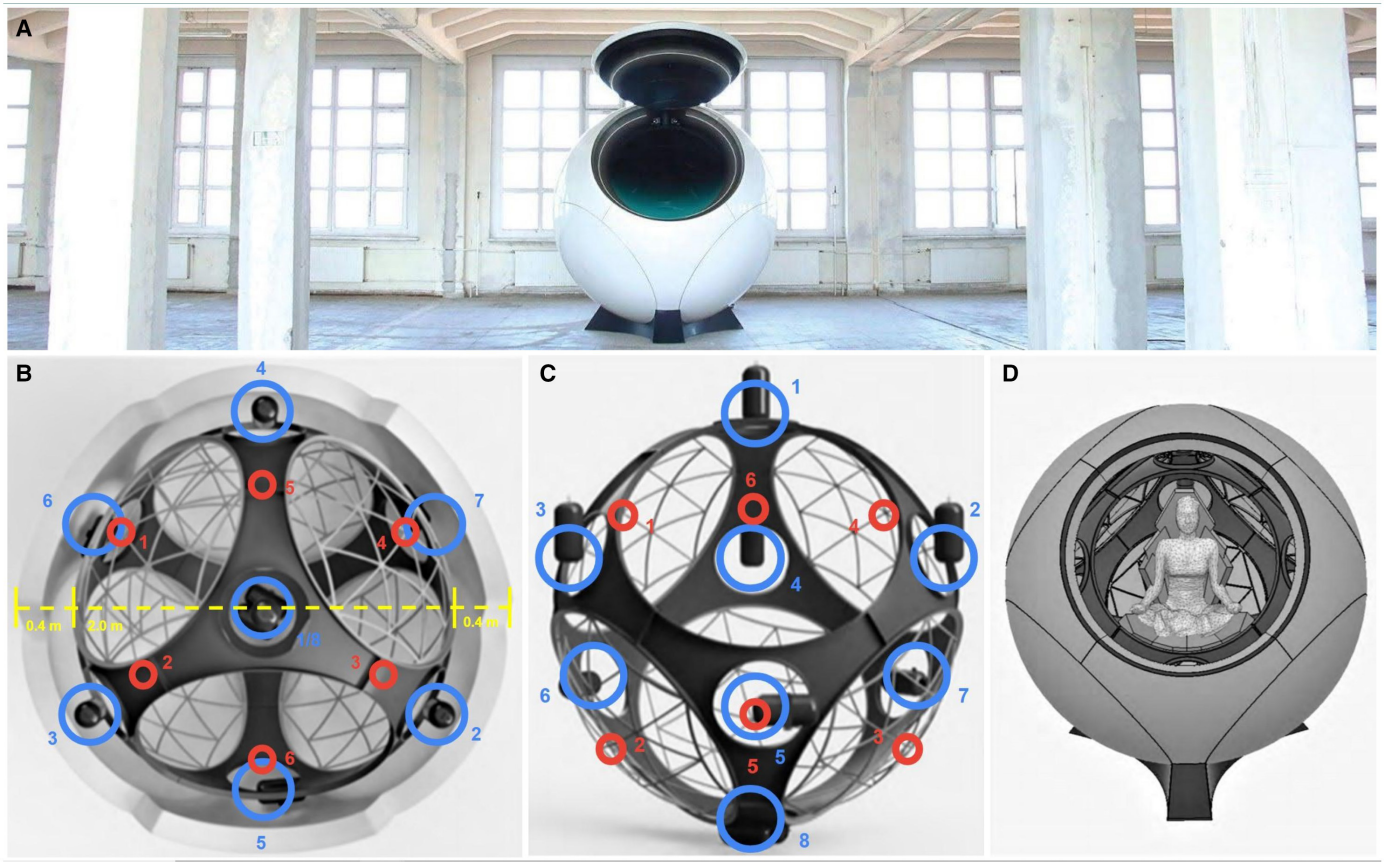
**(A)** The Sphere at The Works Research Institute, Budapest, Hungary. **(B,C)** Audio configuration inside the Sphere. Nine omnidirectional loudspeakers (OmniDrive Pro v2; Bloomline Acoustics B.V., Moerdijk, the Netherlands) are situated within an air gap behind a part sound-transparent, part sound-absorbing inner shell of 2 m minimum inner diameter, encapsulated by a sound-insulating and sound-absorbing outer shell of 2.8 m maximum outer diameter. Measures are indicated in yellow. The loudspeakers are positioned at equal radial angles and equidistant from the center of the Sphere (width, depth, height = 0,0,0; in m). The coordinates of the loudspeakers (Ls) are: Ls1 (0.000, 0.000, 1.000); Ls2 (−0.816, 0.472, 0.333); Ls3 (0.816, 0.472, 0.333); Ls4 (0.000, −0.943, 0.333); Ls5 (0.000, 0.943, −0.333); Ls6 (0.816, −0.472, −0.333); Ls7 (−0.816, −0.472, −0.333); and Ls8/9 (0.000, 0.000, −1.000). Loudspeaker positions and indices are indicated in blue. Six omnidirectional microphones (DPA4060; DPA Microphones A/S, Kokkedal, Denmark) are positioned in each center between four loudspeakers, at equal radial angles and equidistant from the center of the Sphere. The coordinates of the microphones (Mic) are: Mic1 (0.707, −0.408, 0.557); Mic2 (0.707, 0.408, −0.557); Mic3 (−0.707, 0.408, −0.557); Mic4 (−0.707, −0.408, 0.557); Mic5 (0.000, −0.817, −0.557); and Mic6 (0.000, 0.817, 0.557). Microphone positions and indices are indicated in red. **(D)** Subject situated in seated position inside the Sphere.

Physiological and acoustic measurements were obtained inside the Sphere, a bespoke acoustic environment for high-fidelity spatial sound projections (Figures 2A–D). Its applications include single-subject interaction with immersive sound environments for therapeutic, diagnostic and scientific purposes. The Sphere was developed and built by The Works Research Institute between 2017 and 2020, and has since been employed as a test environment to facilitate research and development of new methods and technologies in spatial audio processing and acoustic holography (Oomen, 2021, 2022, 2025; Oomen and Geffen, 2022; Oomen and de Klerk, 2023), in conjunction with acoustic studies on sound field reproduction (Oomen et al., 2026).

## 2 Materials and methods

### 2.1 Acoustic test environment

The Sphere is a sound-proof anechoic spherical enclosure (Figure 2A) with an integrated configuration of eight omnidirectional loudspeakers and six omnidirectional microphones (Figures 2B,C). A custom designed chair to support human subjects in a seated position is centered inside the Sphere (Figure 2D). Environmental noise levels are reduced by ~30 dB when the door of the Sphere is closed. The absorption coefficient of the inner shell of the Sphere is α = 0.99 (*f* > 200 Hz) and of the chair α = 0.90 (*f* > 500 Hz). The maximum permitted uncertainty for acoustic measurements inside the Sphere is *u* = 1.58% (<0.2 dB amplitude and <3 degrees phase shift).

### 2.2 Technical setup

**Figure 3:**
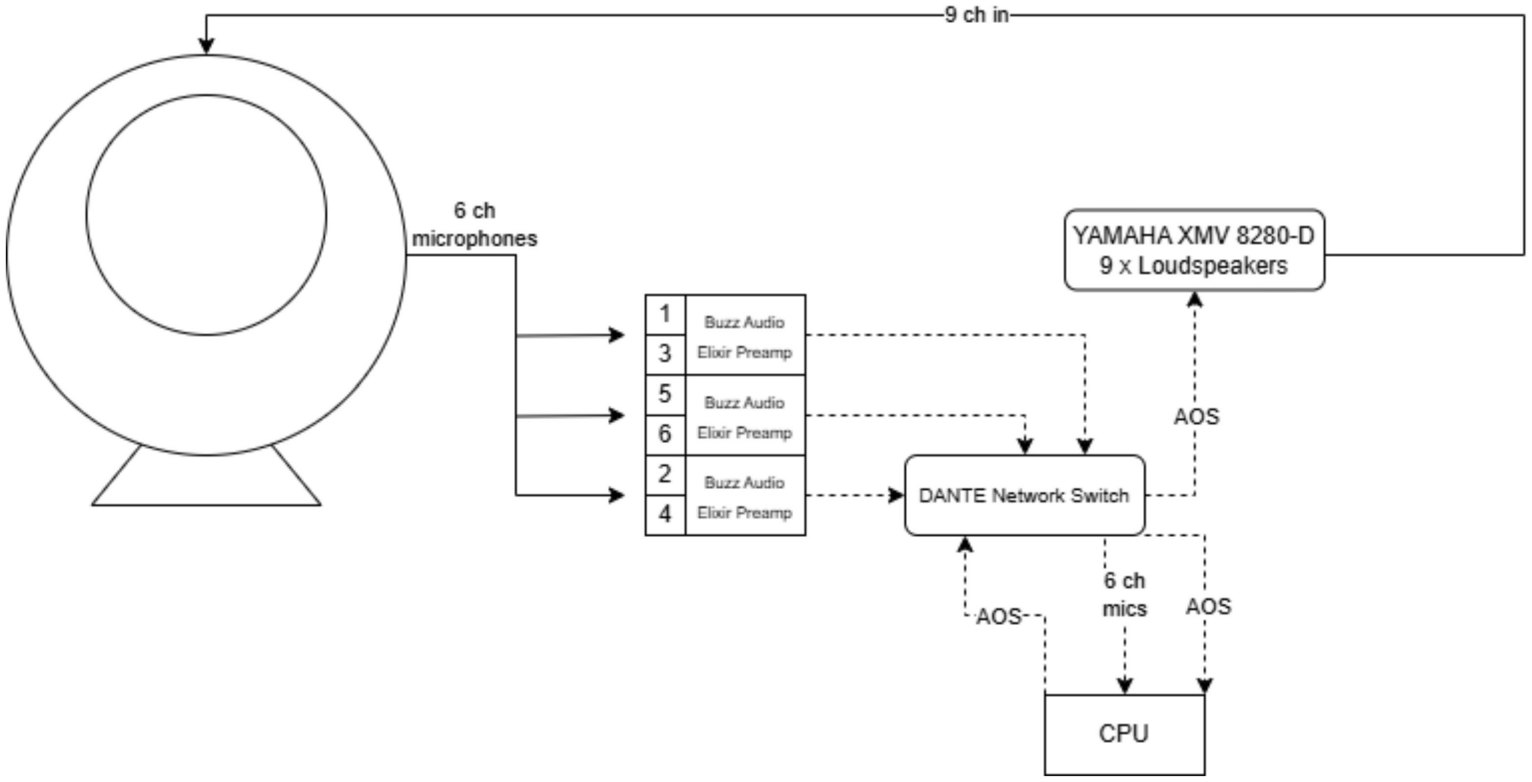
Signal flow of the technical setup. The CPU processes an audio input signal (AIS) distributed to 8 loudspeaker channels (with ch 8 = Ls8/Ls9), comprising the audio signal components of the audio output signal (AOS). The loudspeakers are powered by a DANTE-enabled amplifier (Yamaha XMV8280-D; Yamaha Corporation, Hamamatsu, Japan). Audio signals are distributed over a digital audio network through ethernet (DANTE) at a speed of 1 GB/s with a resolution of 32-bit depth and a sample rate of 48 kHz. Devices are networked using a wireless router (TP-Link Archer C7 AC1750; TP-Link Systems Inc., Irvine, California) and signals are distributed by a 16-Port 1 GB Network Switch (TP-Link TL-SG1016PE; TP-Link Systems Inc., Irvine, California). The clock source of the network is the Yamaha XMV 8280-D amplifier. The 6 microphones are each powered by a preamp (Buzz Audio Elixir True Class A Microphone Amplifier; Buzz Audio Ltd, Blenheim, New Zealand) before A/D conversion. The discrete signals obtained from the microphones are recorded and stored at the CPU. Solid connecting lines represent analog audio signals; dashed connecting lines represent digital signals.

A CPU processes the audio input signal (AIS) and distributes it to eight loudspeaker channels, comprising the audio signal components of the audio output signal (AOS). Acoustic recordings are obtained using six microphones. The total fixed latency from source (CPU) to destination (loudspeakers) is 10 ms, and another 10 ms from receivers (microphones) back to source (Figure 3).

### 2.3 Sound stimuli

**Figure 4:**
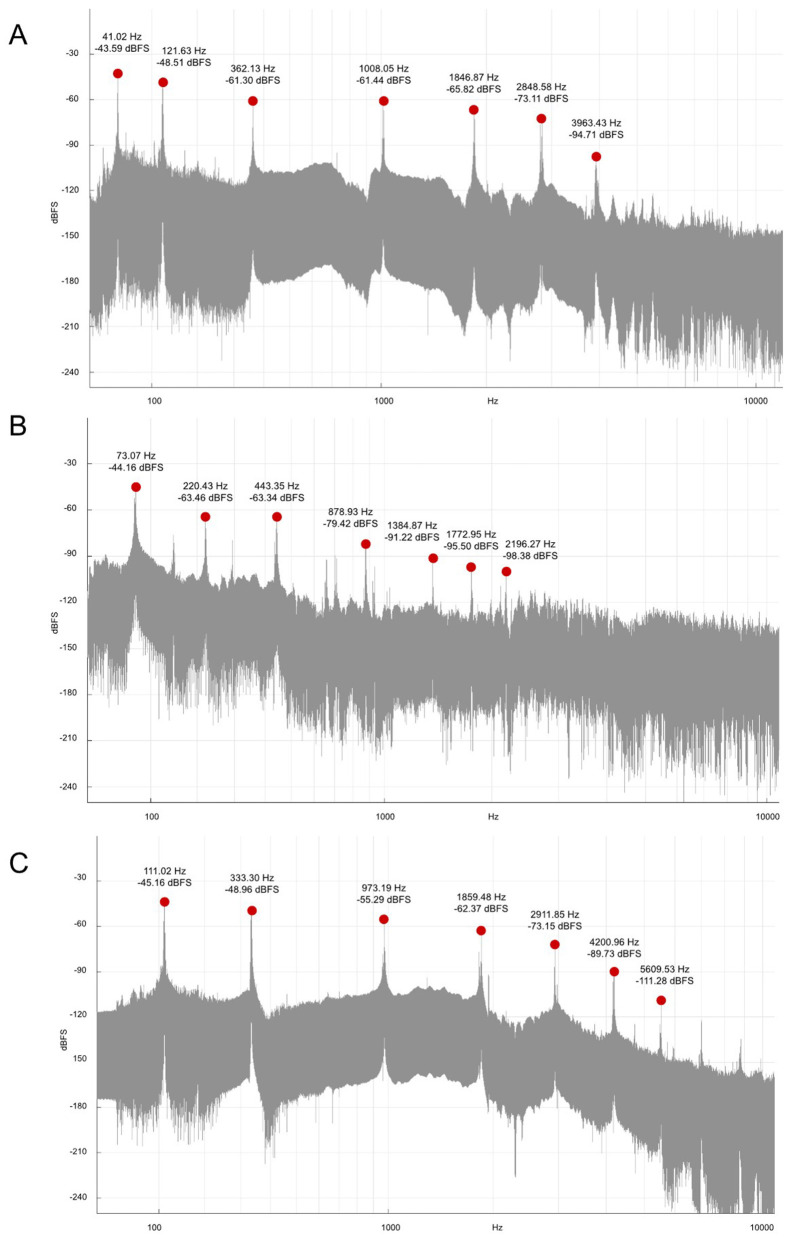
**(A–C)** Frequency spectrum of the 40 Hz, 73 Hz, 110 Hz stimuli (AIS). **(A)** Points of maximum amplitude in the frequency spectrum indicate the most prominent harmonics of the 40 Hz stimulus. **(B)** Points of maximum amplitude in the frequency spectrum indicate the most prominent harmonics of the 73 Hz stimulus. **(C)** Points of maximum amplitude in the frequency spectrum indicate the most prominent harmonics of the 40 Hz stimulus.

The sound stimuli projected inside the Sphere comprise three pre-recorded samples of a singing bowl with a fundamental frequency of 40 Hz, 73 Hz, and 110 Hz, respectively. Each stimulus comprises a single hit of the singing bowl with a total decay time of 16.55 s, 19.56 s, and 14.22 s, respectively. For each experimental trial, a stimulus is repeated to provide a total duration of ~5 min, which corresponds to 17, 15, and 21 repetitions of the sample, respectively. The frequency and peak amplitude of the prominent harmonics of each stimulus are shown in Figures 4A–C.

Spatial projections of the singing bowl sounds were computed using acoustic simulation software (4DSOUND version 2.0) and distributed across the eight loudspeaker channels in the Sphere. Each spatial sound projection consisted of a virtual source and a virtual space, where the virtual source is positioned in the center of the virtual space coinciding with the center of the Sphere (0, 0, 0).

**Figure 5:**
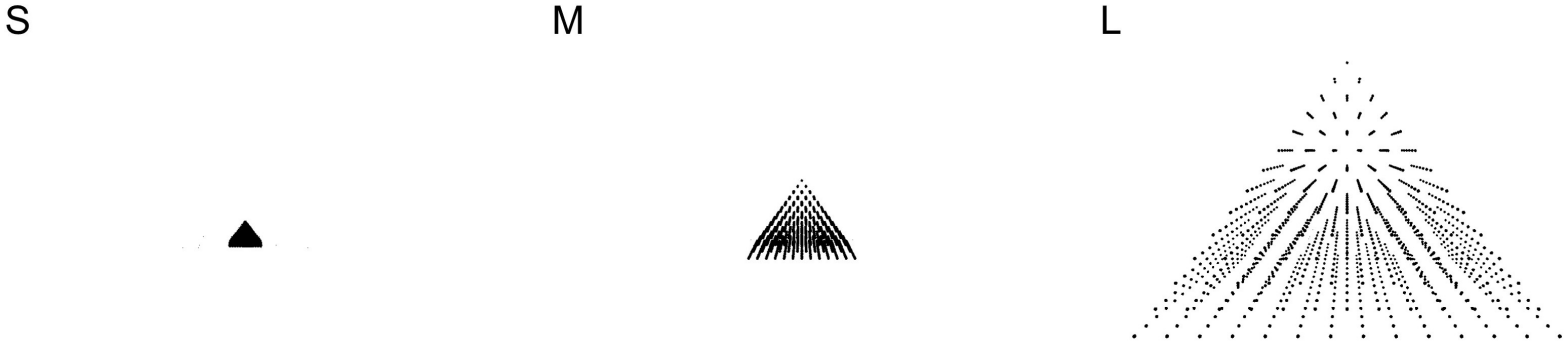
Depiction of a virtual space with the shape of an equilateral pyramid in three room sizes (S, M, L). The virtual space is defined by a plurality of virtual points associated with generated audio signal components, such that a virtual source is perceived by a subject as reverberating within the virtual space at a given position relative to the subject (Oomen, 2022).

The 40 Hz stimulus was projected as reverberating in a virtual room in the shape of a pyramid (C1) in three different sizes: small room size of 2 m base width with a reverberation time (*RT*) = 2 s (S); medium room size of 8 m base width and *RT* = 8 s (M); and large room size of 18 m base width and *RT* = 12 s (L), as shown in Figure 5. The reverberation of the virtual rooms was computed using recursive absorption of the sound energy per octave bands: 67.5 Hz = 0.02; 125 Hz = 0.02; 250 Hz = 0.02; 500 Hz = 0.03; 1k Hz = 0.04; 2k Hz = 0.05; 4k Hz = 0.05; 8k Hz = 0.05; 16k Hz = 0.05.

**Figure 6:**
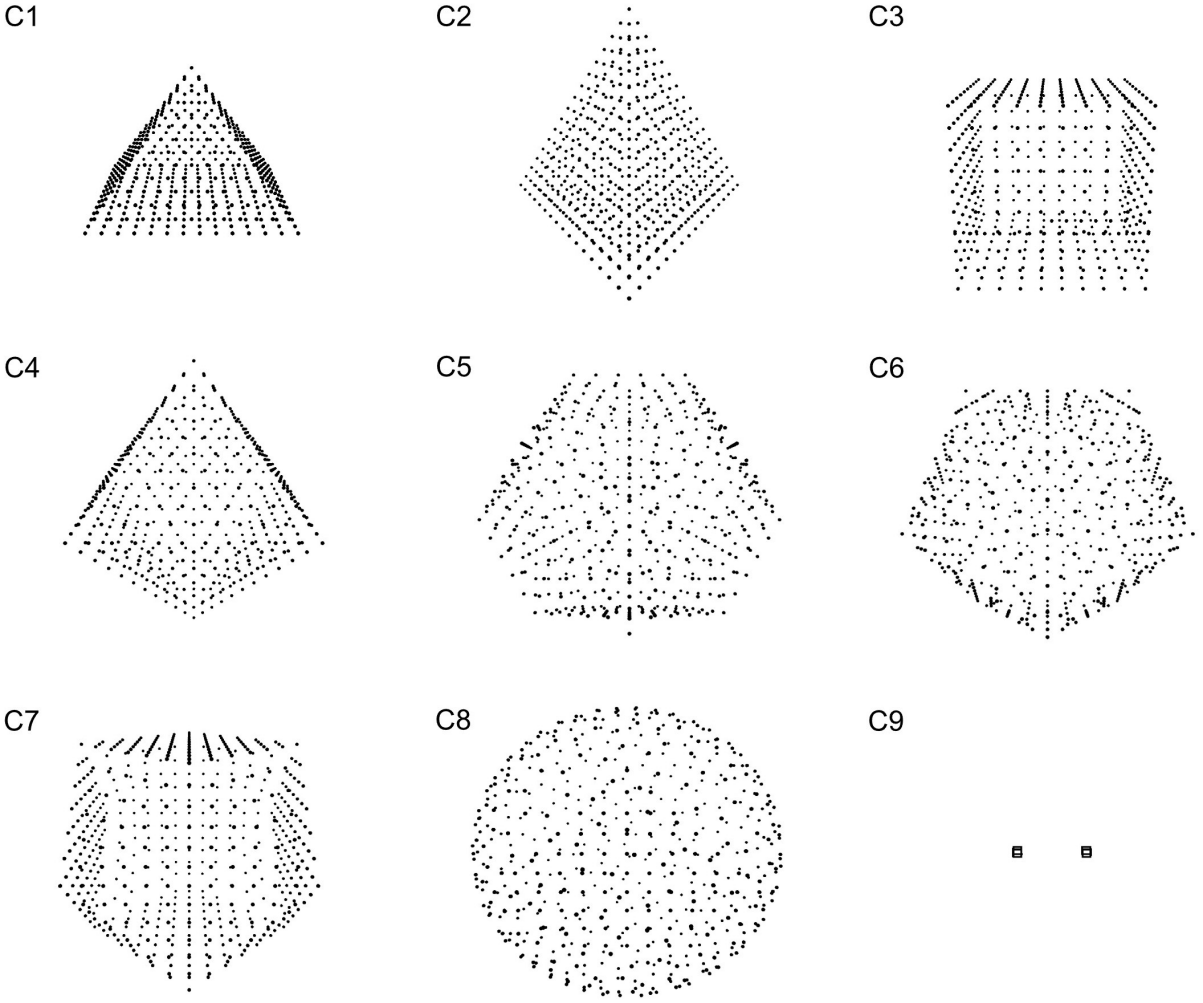
Depiction of virtual spaces with a geometric shape (C1–C9). Each virtual space is defined by a plurality of virtual points associated with generated audio signal components, such that a virtual source is perceived by a subject as reverberating within the virtual space at a given position relative to the subject (Oomen, 2022).

The 73 Hz and 110 Hz stimuli were projected as reverberating in a medium-sized virtual room (8 m base width, *RT* = 8 s) in eight different shapes: pyramid (C1); tetrahedron (C2); cube (C3); octahedron (C4); icosahedron (C5); dodecahedron (C6); cuboctahedron (C7); and sphere (C8). The shapes were chosen as a representative sample of basic three-dimensional geometries. As a ninth condition (C9), the stimuli were projected in stereo, i.e. one channel left (L) and one channel right (R), where the destination of L = Ls2 and R = Ls3 (see Figures 2B,C) without acoustic simulation. The virtual shape configurations are shown in Figure 6.

All sound stimuli were equalized to a maximum sound pressure level (SPL) of ~75 dB. The SPL was decided as a safe level of exposure (World Health Organization, 2022) while maintaining high fidelity of the sound source reproduction. The SPL was determined using a digital sound level meter (BK Precision 732A Digital Sound Level Meter; Brüel and Kjær A/S, Nærum, Denmark) positioned inside the Sphere at the approximate location of the head of the subject, and the amplitude of each AIS was adjusted to obtain the equal SPL. Any other variations in spatial parameters were engineered to accommodate optimal sound performance per frequency while maintaining the highest performance proximity between conditions.

### 2.4 Participants

A total of 59 subjects (26 male; 33 female) participated in the study. Participants were 18 years or older; healthy; sober; proficient in English; not pregnant; with normal hearing; did not have an artificial heart pacemaker, cerebral shunt and/or prosthetic joints; and had no history of neurological or psychiatric disease, e.g., no seizures, epilepsy (especially sound-induced epilepsy), claustrophobia or other issues that could interfere with the valid interpretation of results.

Participants provided written and informed consent prior to enrollment in the study. Trials with human subjects were conducted on site at The Works Research Institute in Budapest, Hungary, in accordance with the Declaration of Helsinki and approved by The United Ethical Review Committee for Research in Psychology (EPKEB). Habitual caffeine intake and daily dietary habits were permitted throughout the study. Participants received a small reimbursement for providing their time and covering travel expenses.

### 2.5 Experimental procedure

Subjects (*n* = *59*) who met the entry criteria were enrolled in a consecutive order and were divided into three groups: 15 subjects (7 male; 8 female) were exposed to the 40 Hz stimulus; 26 subjects (16 female; 10 male) were exposed to the 73 Hz stimulus; and, 18 subjects (9 female; 9 male) were exposed to the 110 Hz stimulus. Within each group, subjects were exposed to blocks of stimuli in different conditions as shown in Table 1. The order of stimulus conditions was randomly alternated between subjects and recorded in a single blind paradigm.

**Table 1:** Blocks of sound stimuli within each subject group.

Group	Condition name	Room size	Shape
40 Hz (*n =* 15)	S	Small	Pyramid
	M	Medium	Pyramid
	L	Large	Pyramid
73 Hz (*n =* 26), 110 Hz (*n =* 18)	C1	Medium	Pyramid
	C2	Medium	Tetrahedron
	C3	Medium	Cube
	C4	Medium	Octahedron
	C5	Medium	Icosahedron
	C6	Medium	Dodecahedron
	C7	Medium	Cuboctahedron
	C8	Medium	Sphere
	C9		Stereo

Stimulus conditions are organized according to frequency, room size, and shape.

Examination of each group was executed sequentially. First, subjects in the 40 Hz group were examined across three conditions of room size with the same room shape to determine the effects of room size on the subjects. Then, subjects in the 73 Hz and 110 Hz groups were examined across nine conditions of room shape with the same room size to determine the effects of room shape (within-group differences). This study design also allowed determining discrete effects of stimulus frequency regardless of spatial conditions (between-group differences) and discrete effects of room shapes regardless of stimulus frequency (between-condition differences). Either room size or room shape was assessed within one group to reduce variables in the experimental procedure. The association of frequency and spatial conditions within each group was arbitrary.

Subjects were comfortably seated in a fixed position supported by the chair, with their sternum approximately located at the center of the Sphere (see Figure 2D). All subjects were seated in an identical position in complete darkness and were requested to close their eyes and not to move during the trial. To determine functional connectivity of the auditory cortex for each subject, a 500 Hz test tone of 10 ms was presented 200 times at a repetition rate of 0.75 s in a pseudo-random order from the left and right speakers (Ls2, Ls3) on the horizontal plane at ear level. A resting state condition (baseline) was then recorded for each subject with eyes closed in complete silence for 5 min. After this presentation, subjects were exposed to the respective stimuli blocks each lasting 5 min.

### 2.6 Outcome measures

#### 2.6.1 Profile of mood states and multidimensional mood questionnaire

To assess psycho-emotional response to the sound stimuli, subjects were monitored before and immediately after the experimental procedure by means of the Profile of Mood States Second Edition (POMS2) and the Multidimensional Mood Questionnaire (MDMQ), administered in this respective order. The POMS2 is a validated psychological test for mood assessment, suitable for individuals aged 13 and older (Heuchert and McNair, 2012; Nyenhuis et al., 1999). The POMS2 was used in the short version for adults, consisting of 35 items divided into 5 response categories (scored from 0 to 4). The MDMQ is a widely used and thoroughly validated questionnaire to assess mental state (Steyer et al., 1994). The English version was used and comprises 30 items divided into 5 response categories (scored from 1 to 5).

Data analysis was performed using statistical software (IBM SPSS version 29.0.0). A paired-sample *t*-test was used to compare the means of pre and post-scores for each item in the POMS2 and MDMQ and reported as mean values and standard error of the mean (SEM) with a statistical significance threshold set at *p* < 0.05.

#### 2.6.2 Electroencephalography

EEG was recorded using 64 channels of the EEG cap (g.Nautilus Research 64 ch; g.tec medical engineering GmbH, Schiedlberg, Austria). The distance between loudspeakers inside the Sphere and the head of the subject was ~0.67–1.33 m. Electrodes of the EEG cap were shielded and subjects were grounded via an electrode to the EEG amplifier to minimize electrical interference. Prior to the experimental procedure, the EEG setup was tested inside the Sphere with and without a subject and no systematic interference was identified.

Analysis was conducted at the sensor level providing topographical information on brain activity, involving three steps: (i) data pre-processing, (ii) frequency analysis and (iii) statistical analysis. All EEG data was analyzed in MATLAB using FieldTrip (Oostenveld et al., 2011).

Continuous EEG recordings were segmented in trials of 1 s time length. After segmentation, trials were baseline corrected and a notch filter was applied to remove 50 Hz, 100 Hz, and 150 Hz power line noise. Trials and channels were then visually inspected for artifacts and the ones with excessively high variance were rejected. Furthermore, an independent component analysis was applied and components were visually inspected. Components representing eye movements, heart beat activity and muscular artifacts were rejected. After artifact cleaning, previously rejected channels were recovered by interpolation. Finally, channels were re-referenced to a common average.

A Fourier analysis in the range from 4 to 100 Hz in steps of 2 Hz was computed by implementing a multitaper approach using Hanning tappers. EEG power was then averaged across trials separately for each stimulus sorted by frequency and condition. In order to assess significant differences among conditions, a cluster-based nonparametric permutation test with a cluster-alpha of α = 0.05 and minimum cluster size of 2 neighboring channels was implemented. These comparisons allowed for the evaluation of significant differences between conditions in any given frequency bin and for any group of EEG channels.

#### 2.6.3 Heart rate and heart rate variability

The electrical activity of the heart was measured via a Re-usable Pulse Oximetry Sensor (EnviteC-Wismar GmbH; Wismar, Germany) placed on the distal phalange of the index finger of the right hand of the subject and recorded via the EEG cap using multifunctional electrodes which can be alternated to monitor physiological responses non-invasively. The recorder provided the instantaneous HR at a sampling rate of 250 Hz and was used to continuously record the HR during the presentation of sound stimuli. The continuously recorded signals were then segmented into trials starting with the onset of the sound stimuli presented.

From the HR recordings the following parameters were derived: (i) mean HR for each trial; (ii) variation of HR as the standard deviation of heart beat frequency within individual trials. To capture the dynamics of the HR during the repeated presentation of individual sounds, the slope and intercept of linear regression of the stimulus repetitions on both the mean and the variation of HR were determined. The intercept indicates the HR at the start of each trial, while the slope indicates the tendency of HR to increase or decrease during the trial.

In an additional step, the HRV was determined using (Equation 1).


$$\begin{array}{c} HRV\;=\;\sqrt{\overset{n}{\underset{i=1}{\sum }}{\left(\frac{1}{f\left(p_{i}\right)}\right)}^{2}-\;n\;{\left[\overset{n}{\underset{i=1}{\sum }}\left(\frac{1}{f\left(p_{i}\right)}\right)\right]}^{\;2\;}} \end{array}$$ (1)

with the momentary heart rate at sampling point *p*_*i*_:*f*(*p*_*i*_) with current time point $t_{i}\;=\frac{p_{i}}{s}$ with *s* being the sampling rate, and each next sample $p_{i+1}=p_{i}+\frac{s}{f\left(p_{i}\right)}$.

For this purpose, the RR interval was determined from the HR activity. Furthermore, the sampling point for each subsequent RR interval was also derived. For the calculation of the HRV for a single stimulus type and condition, all trials of the corresponding segment were used.

Although the order of conditions were balanced across subjects, sequence effects might have disturbed any relation between stimulus condition and HRV. Using a general linear model approach by which stimulus condition was modeled by one variable and stimulus order by another, for a single subject *i* the heart rate variability *Z*_*j*_ for stimulus condition *j* is explained by (Equation 2).


$$\begin{array}{c} Z_{j}=\beta X\;+\alpha Y\;+\alpha_{i}+\varepsilon \end{array}$$ (2)

Removing the effect of stimulus order and the individual bias, the correlation between stimulus condition and HRV was assessed with (Equation 3).


$$\begin{array}{c} Z-\;\alpha Y-\;\alpha_{i}=\beta X+\varepsilon \end{array}$$ (3)

#### 2.6.4 Electromyography

EMG was recorded using two channels of the EEG cap as multifunctional electrodes. In order to obtain a strong and easily accessible EMG signal measuring muscular tension and relief, we recorded from the right arm muscle with the EMG1 sensor positioned closer to the shoulder (proximal) and the EMG2 sensor closer to the hand (distal) of the subject.

A notch filter was applied to remove power line noise at 50 Hz on the EMG1 channel. After segmentation, trials were visually inspected for spike-like responses in the signal caused by jerk movements of the subjects and rejected if necessary. A pairwise statistical comparison was performed on the means across all subjects for EMG channels 1 and 2 sorted by stimulus order. Stimulus order was then treated as a covariate and removed from the data using regression analysis.

#### 2.6.5 Galvanic skin response

GSR was measured with a g.GSRsensor2 and was recorded via the EEG cap using multifunctional electrodes. GSR was recorded by attaching two electrodes to the distal phalanges of the index and middle finger of the left hand.

The GSR amplitude was modeled by a line to capture the level and slope for each tone. The variance of the GSR amplitude was taken as a measure for fluctuations of the GSR signal. A spectral analysis was performed on the GSR amplitude. For determining the frequency of the fluctuations, the center of gravity of the frequency was estimated from the spectrum. A second order polynomial fitting was applied to the average center frequency for each condition to capture any changes across time within a trial. The total power and variance across frequencies were also derived. A pairwise statistical comparison was performed on the means across all subjects for the GSR low, mid and high frequencies (frequency parameters 1–3) sorted by stimulus order. Stimulus order was then treated as a covariate and removed from the data using regression analysis.

#### 2.6.6 Acoustic measurements

Acoustic measurements were performed to determine acoustic deviations in the sound stimuli dependent on the subject and stimulus condition. A reconstruction of the scattered wave field around the subject was obtained in two steps: (i) acquiring audio signals from the spatial microphone array (see Figures 2B,C); and (ii) signal processing to obtain sound wave deviations at discrete frequencies and spatial positions.

Continuously recorded audio signals were segmented into trials starting with the onset of each sound stimulus. Trials were exported as stand-alone WAV files with identical time length, encoded at a resolution of 32-bit depth and a sample rate of 48 kHz. During the recording of each trial, unforeseen movements and/or noise produced by the subjects was logged and the corresponding time fractions in the recordings were eliminated prior to analysis, and later recovered by interpolation.

Frequency analysis was performed by means of a fast Fourier transform (FFT) of each microphone signal corresponding to a spatial position inside the Sphere for the total duration of each segmented trial. The maximum amplitude *L*_0_ was logged from the FFT for each of the prominent harmonic frequencies (see Figures 4A–C). Each trial with a subject in the acoustic environment was then compared to a reference trial of the corresponding stimulus without a subject (REF) to derive the amplitude deviation and phase shift for each frequency and spatial position (Equations 4–10).

Let two compared signals with the same frequency (*s*_1_, *s*_2_ = *REF*) be expressed as


$$\begin{array}{c} s_{1}=A_{1}\;sin\left(\omega t-\;\phi_{1}\right)\\ s_{2}=A_{2}\;sin\left(\omega t-\phi_{2}\right) \end{array}$$ (4)

where the amplitude $A=\;10^{\;\frac{L_{0\;}}{20}}\;and\;\phi$ is the phase of each signal. The amplitude deviation *A*_*D*_ (in dB) between the two signals is determined as


$$\begin{array}{c} A_{D}=L_{1}-L_{2} \end{array}$$ (5)

The second signal is then shifted by 180 degrees and added to the first signal, i.e., their difference is determined as


$$\begin{array}{c} s_{D}=s_{1}-s_{2}=A_{1}\;sin\left(\omega t-\phi_{1}\right)-A_{2}\;sin\left(\omega t-\phi_{2}\right) \end{array}$$ (6)

This function is harmonic with the same frequency, but a new amplitude α and phase β as follows:


$$\begin{array}{c} s_{D}=\alpha \;sin\left(\omega t-\beta \right) \end{array}$$ (7)

The phase shift between the two signals is then determined as


$$\begin{array}{c} \psi \;=arccos\;\frac{A_{1}^{2}\;+\;A_{2}^{2}\;-\;\alpha^{2}}{2\;A_{1}\;A_{2}} \end{array}$$ (8)

It should be noted that arccos returns a value for ψ between 0 and π;, thus, the sign of ψ cannot be determined this way. The second signal is then shifted by 90 degrees using a Hilbert transform and added to the first signal, and their difference is taken a second time as


$$\begin{array}{c} s_{D\;2}=s_{1}-s_{2}=A_{1}\;sin\left(\omega t-\phi_{1}\right)-A_{2\;}sin\left(\omega t-\phi_{2}+\frac{\pi }{2}\right) \end{array}$$ (9)

and ψ_2_ is obtained as


$$\begin{array}{c} \psi_{2}=arccos\;\frac{A_{1}^{2}\;+\;A_{2}^{2}\;-\;\alpha_{2}^{2}\;}{2\;A_{1}\;A_{2}} \end{array}$$ (10)

The sign of ψ is then determined as −ψ if $\psi_{2}>\frac{\pi }{2}\;and\;+\psi \;$if $\psi_{2}<\frac{\pi }{2}$.

Finally, the variability of deviations was assessed by determining the average difference of each consecutive trial *t*_*i*_ to the next *t*_*i*+1_ as defined in (Equation 11).


$$\begin{array}{c} Variability\;=\;\frac{\sum_{i=1}^{n}\sqrt{{\left(t_{i}\;-t_{i+1}\right)}^{2}}}{n} \end{array}$$ (11)

### 2.7 Statistical analysis

A pairwise statistical comparison was performed on the adjusted means of each outcome measure sorted by stimulus frequency and condition. By sorting each outcome measure by frequency, any main effects between groups were inferred, regardless of stimulus order and condition. Sorting the outcome measures by condition enabled inference of any significant differences in the physiological expression of subjects related to the spatial condition, i.e. the room size and/or shape. Unadjusted means were sorted by stimulus order to infer any significant differences in the physiological expression of subjects related to stimulus duration, regardless of frequency and condition. Statistical significance of the differences for each outcome measure was determined with a two-sample independent *t-*test and reported as two-tailed *p-*values with a significance level of α = 0.05. To obtain the *t-*test, the standard deviation of the sample mean was used to estimate the SEM.

Significant correlations between outcome measures are reported after determining the Pearson correlation coefficient (*R*) between acoustic deviations at a given frequency *f* and a change in physiological expression *BioM* for the corresponding conditions. By determining physioacoustic correlations, any acoustic factors that mediated the observed physiological expression of subjects could be inferred, i.e., pertaining to the amplitude deviation and phase shift per frequency across spatial positions and segmented trials. Significant correlations are reported as two-tailed *p-*values with a significance level of α = 0.05 adjusted using the Bonferroni correction, depending on the number of comparisons performed in parallel.

## 3 Results

### 3.1 POMS2 and MDMQ

**Figure 7:**
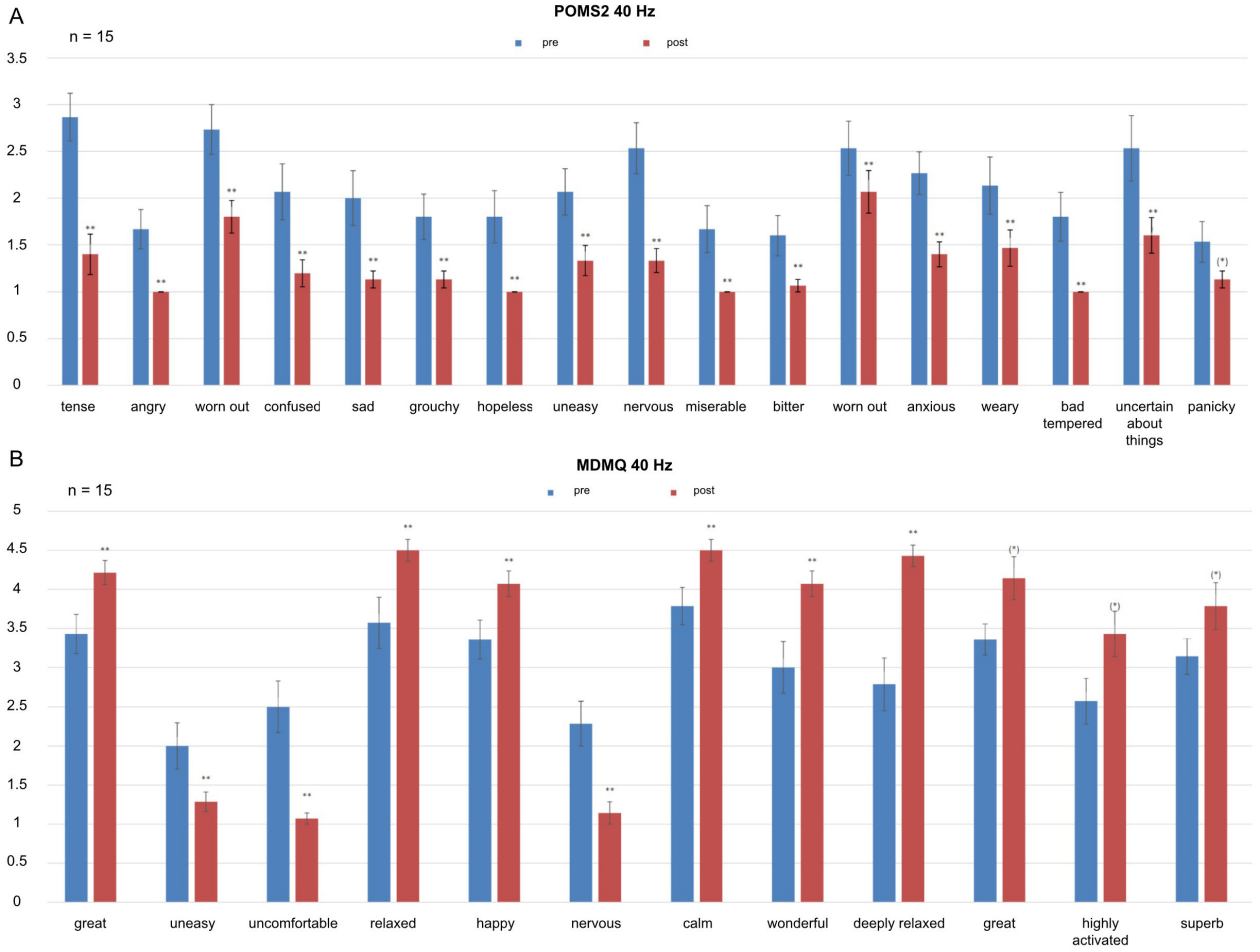
**(A, B)** Mean and standard error of items from the POMS2 and MDMQ with significant differences pre and post-exposure to the 40 Hz stimulus (*n* = 15). Highly significant differences (*p* < 0.005) are indicated with **, marginally significant differences (*p* = 0.05) are indicated with (*).

**Figure 8:**
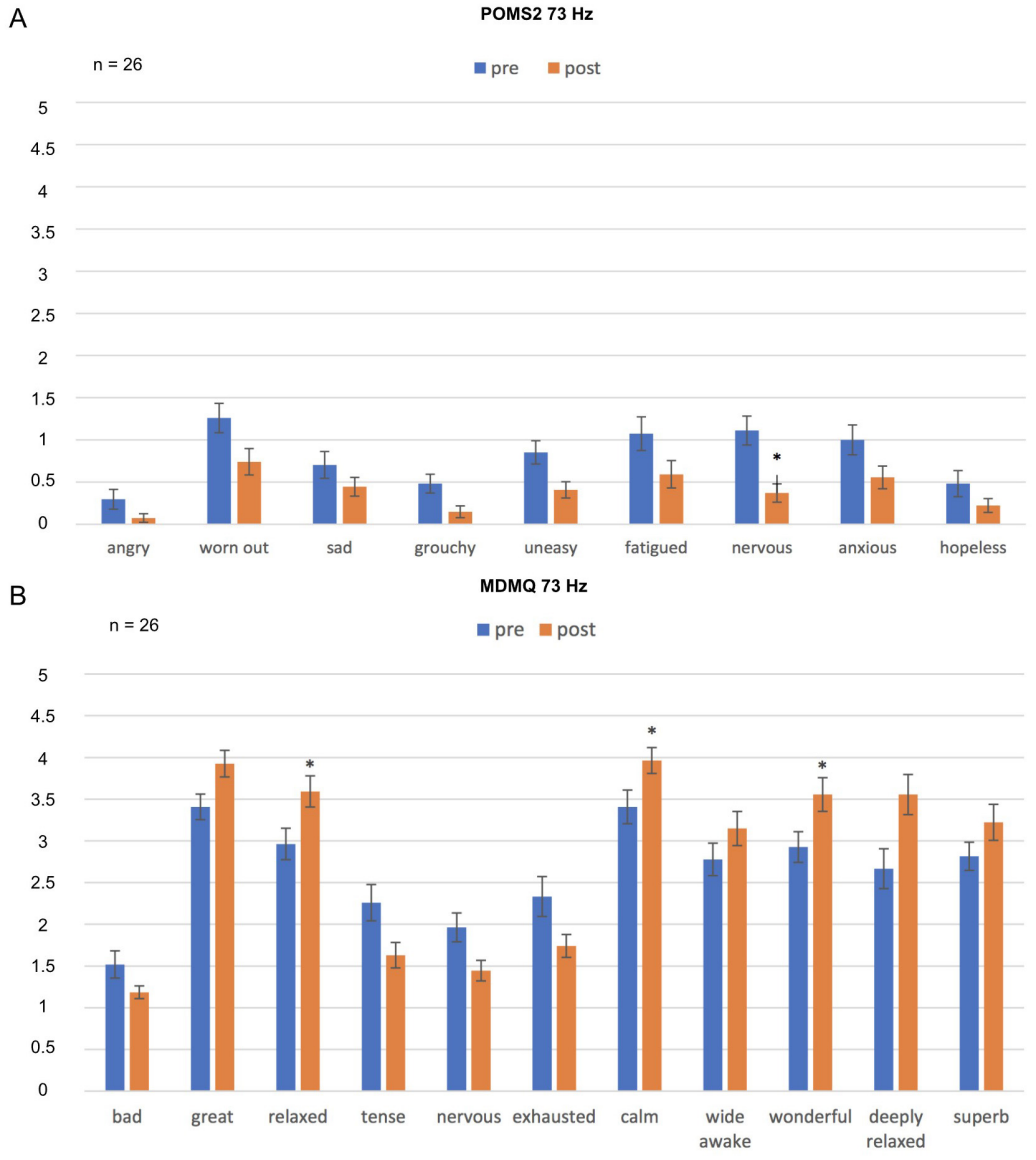
**(A, B)** Mean and standard error of items from the POMS2 and MDMQ with significant differences (*p* < 0.05) pre and post-exposure to the 73 Hz stimulus (*n* = 26). Highly significant differences (*p* < 0.005) are indicated with *.

**Figure 9:**
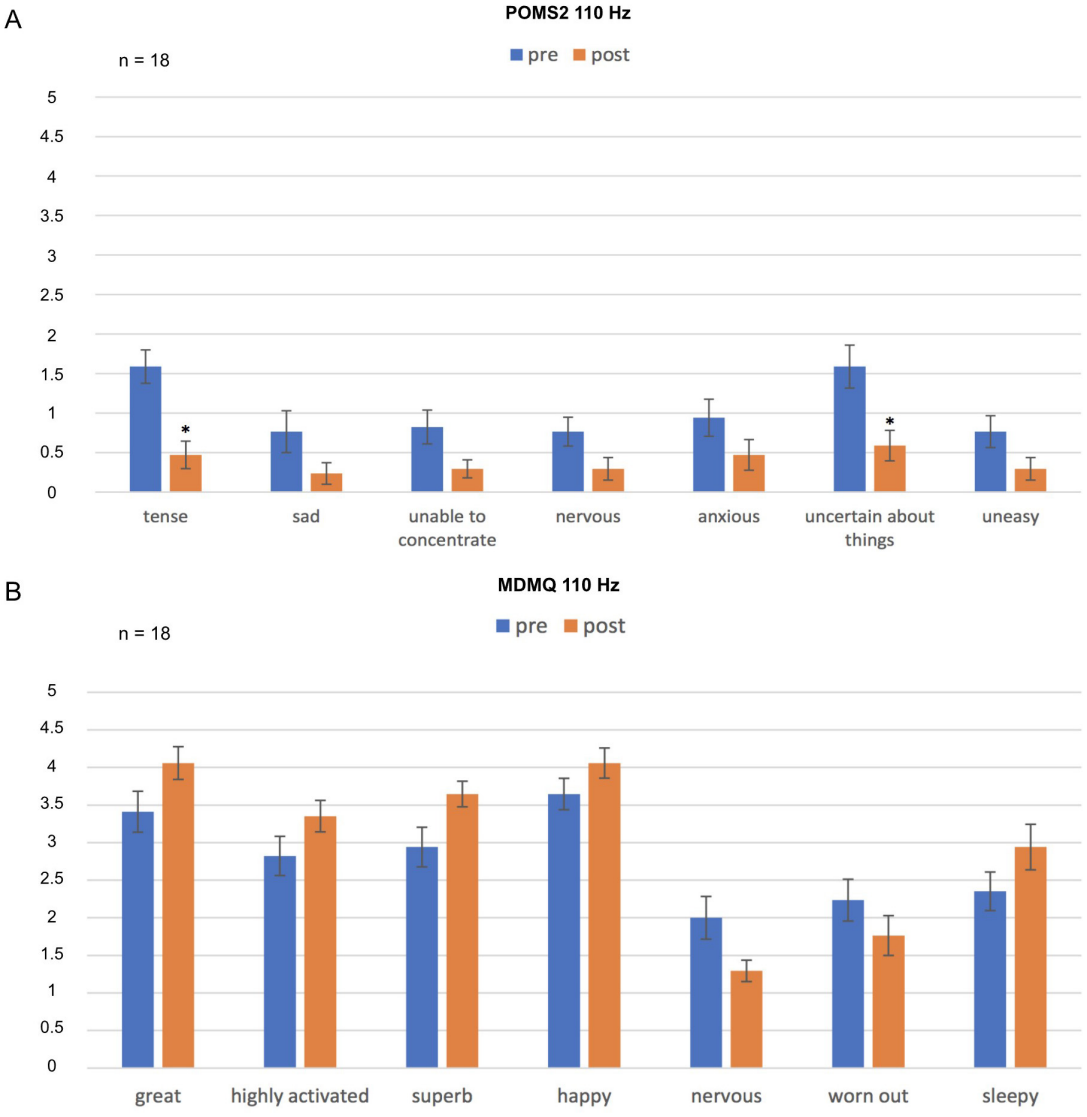
**(A, B)** Mean and standard error of items from the POMS2 and MDMQ with significant differences (*p* < 0.05) pre and post-exposure to the 110 Hz stimulus (*n* = 18). Highly significant differences (*p* < 0.005) are indicated with *.

Significant differences in items from the POMS2 and MDMQ pre and post exposure to the 40 Hz, 73 Hz and 110 Hz stimuli are shown in Figures 7A, B, 8A, B, 9A, B, respectively.

### 3.2 EEG

**Figure 10:**
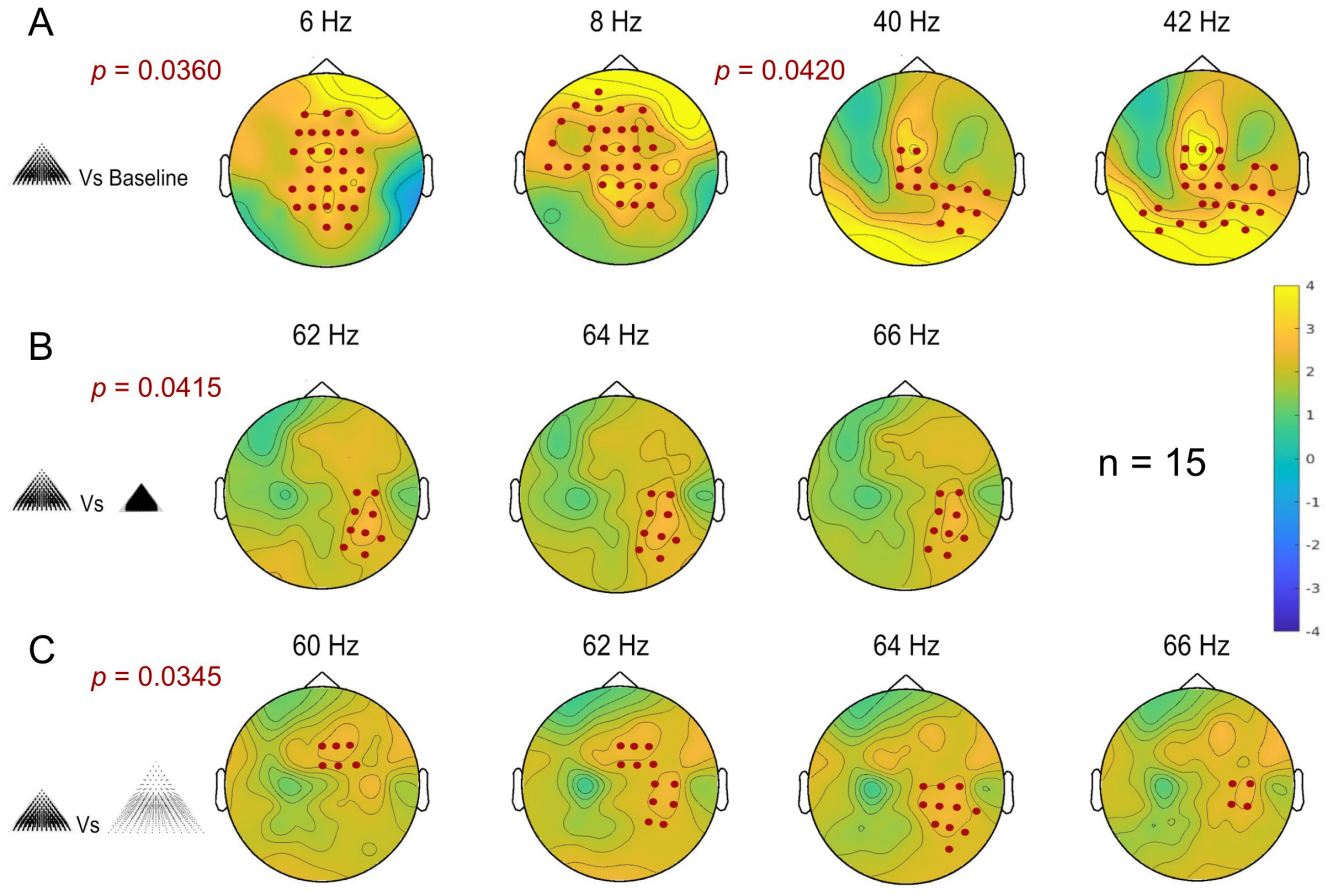
**(A–C)** Significant differences in EEG power as distributed across the brain scalp at respective frequencies for the 40 Hz stimulus (*n* = 15) projected in different room sizes. **(A)** Increased activation in a medium room size (M) compared to baseline. Frontal, parietal and occipital channels show a significant increase in activity from 6 to 8 Hz. Parietal, right temporal and occipital channels show a significant increase in activity from 40 to 42 Hz. **(B)** Increased activation in a medium room size (M) compared to a small room size (S). Right temporal and occipital channels show a significant increase in activity from 62 to 66 Hz. **(C)** Increased activation in a medium room size (M) compared to a large room size (L). Frontal, right temporal and occipital channels show a significant increase in activity from 60 to 66 Hz. The extension of significant clusters across electrodes is marked by the red dots. EEG power is indicated as ± microvolts squared (μV^2^).

**Figure 11:**
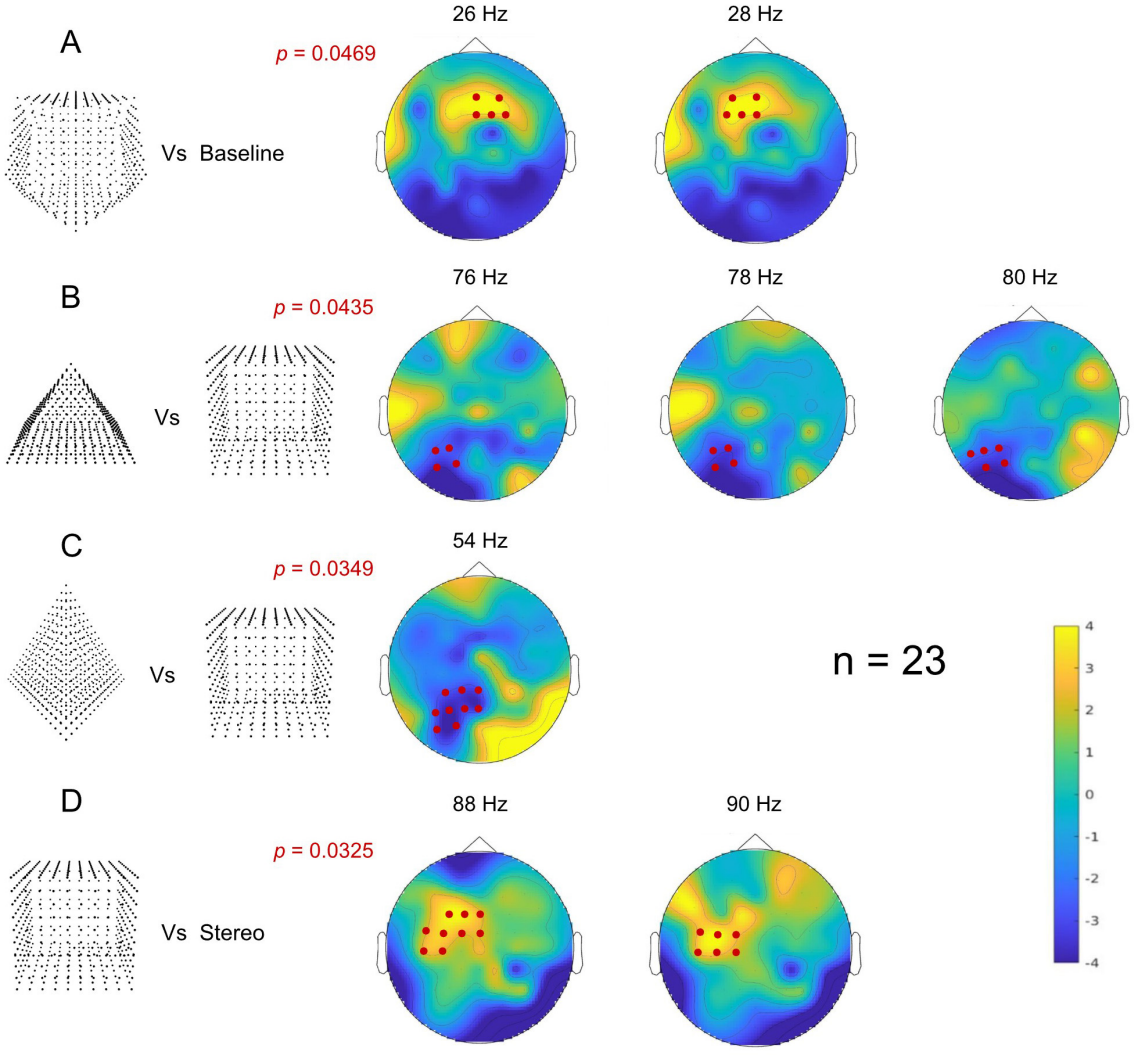
**(A–D)** Significant differences in EEG power as distributed across the brain scalp at respective frequencies for the 73 Hz stimulus (*n* = 23) projected in different room shapes. **(A)** Increased activation in a cuboctahedron (C7) compared to baseline. Frontal channels show a significant increase in activity from 26 to 28 Hz. **(B)** Decreased activation in a pyramid (C1) compared to a cube (C3). Left occipital channels show a significant decrease in activity from 76 to 80 Hz. **(C)** Decreased activation in a tetrahedron (C2) compared to a cube (C3). Left occipital and temporal channels show a significant decrease in activity at 54 Hz. **(D)** Increased activation in a cube (C3) compared to stereo (C9). Left temporal and frontal channels present an increased activation from 88 to 90 Hz. The extension of significant clusters across electrodes is marked by the red dots. EEG power is indicated as ±microvolts squared (μV^2^).

**Figure 12:**
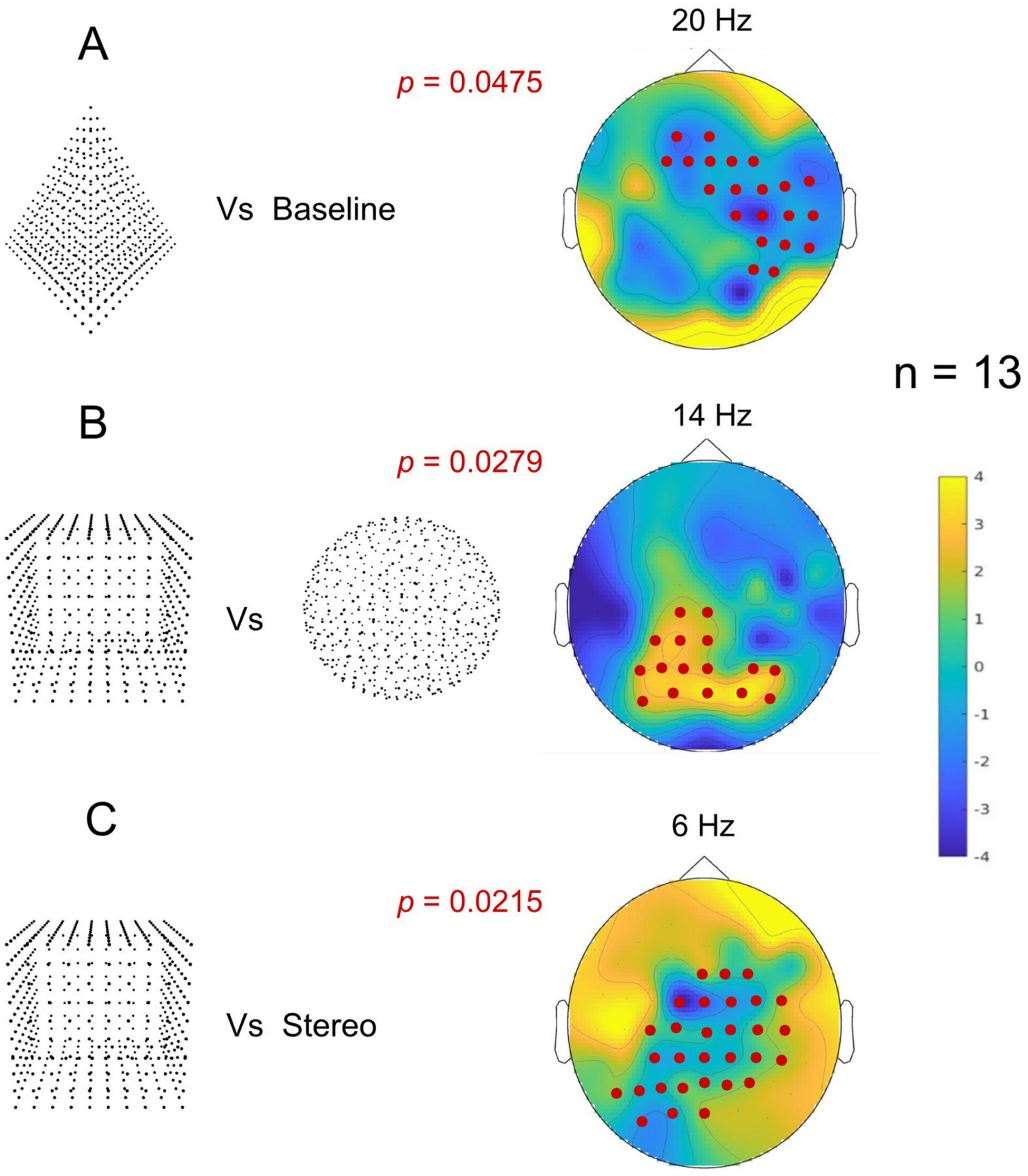
**(A–C)** Significant differences in EEG power as distributed across the brain scalp at respective frequencies for the 110 Hz stimulus (*n* = 13) projected in different room shapes. **(A)** Decreased activation in a tetrahedron (C2) compared to baseline. Right temporal and frontal channels show a significant decrease in activity at 20 Hz. **(B)** Increased activation in a cube (C3) compared to a sphere (C8). Occipital and left temporal channels show a significant increase of activity at 14 Hz. **(C)** Decreased activation in a cube (C3) compared to stereo (C9). Occipital, temporal and frontal channels show a significant decrease in activity at 6 Hz. The extension of significant clusters across electrodes is marked by the red dots. EEG power differences are indicated as ±microvolts squared (μV^2^).

Significant differences in EEG power depending on the room size of the 40 Hz stimulus are shown in Figures 10A–C. Significant differences in EEG mean power depending on the shape of the 73 Hz stimulus and 110 Hz stimulus are shown in Figures 11A–D, 12A–C, respectively.

Significant differences in localized EEG power between conditions were positively correlated to the localized amplitude deviation of sound waves at discrete harmonic frequencies. The correlation was found to be significant after Bonferroni correction (*R* = 0.92, *p* < 0.00006). The coupling between discrete frequencies of EEG activity and sound waves was found to be dependent on the measured phase shift of the sound frequency and determined using (Equation 12).


$$\begin{array}{c} f_{brain}=f_{sound}\;|\;\beta \;\left[\frac{1/f_{sound}}{2\pi }\;\psi \right]+\varepsilon \;| \end{array}$$ (12)

The coupling of EEG and sound frequencies was positively correlated to the phase shift of the sound frequency, and was found to be significant after Bonferroni correction (*R* = 0.87, *p* < 0.00049). The correlated means of EEG power, amplitude deviation and phase shift per frequency are shown in Table 2.

**Table 2:** Localization, frequency range, and mean power of significant differences in EEG between conditions (as shown in Figures 10A–C, 11A–D, 12A–C); and the coupled frequency, mean amplitude deviation (*A*_*D*_) and phase shift (ψ) of sound waves as measured at the microphones in corresponding spatial locations (see Figures 2B,C).

Group	Condition	EEG			Sound				
		Localization	Frequency (Hz)	Mean power (μV^2^)	Localization	Mics	Frequency (Hz)	Mean *A_*D*_* (dB)	Mean ψ (°)
40 Hz	M–baseline	Frontal—parietal—occipital	6–8	2.42	Center	1, 2, 3, 4	41.02	2.10	42.45
40 Hz	M–baseline	Parietal—right temporal—occipital	40–42	2.35	Center—right back	1, 2, 4	362.13	6.59	59.91
40 Hz	M–S	Right temporal—occipital	62–66	1.87	Right back	1	1,846.87	5.73	24.68
40 Hz	M–L	Frontal—right temporal—occipital	60–66	1.99	Front—right back	1, 2	2,848.58	4.03	13.87
73 Hz	C7—baseline	frontal	26–28	2.30	Front	6	443.35	2.22	17.48
73 Hz	C1—C3	Left occipital	76–80	−3.39	Left back	4	1,772.95	−3.07	43.93
73 Hz	C2—C3	Left occipital—temporal	54	−2.77	Left back	4	1,384.87	−3.78	27.33
73 Hz	C3—C9	Left temporal—frontal	88–90	3.07	Center-left front	3, 4, 6	1,772.95	4.66	63.47
110 Hz	C2—baseline	Right temporal—frontal	20	−3.24	Right front	2	973.19	−5.35	14.32
110 Hz	C3—C8	Occipital—left temporal	14	3.36	Center back	1, 2, 3, 4, 5	2,911.85	2.29	38.58
110 Hz	C3—C9	Occipital—temporal—frontal	6	−4.46	Front—center—left back	4, 5, 6	1,859.48	−5.810	12.39

*A*_*D*_is indicated as ±dB. ψ is indicated as degrees (0–180°).

### 3.3 HR and HRV

**Figure 13:**
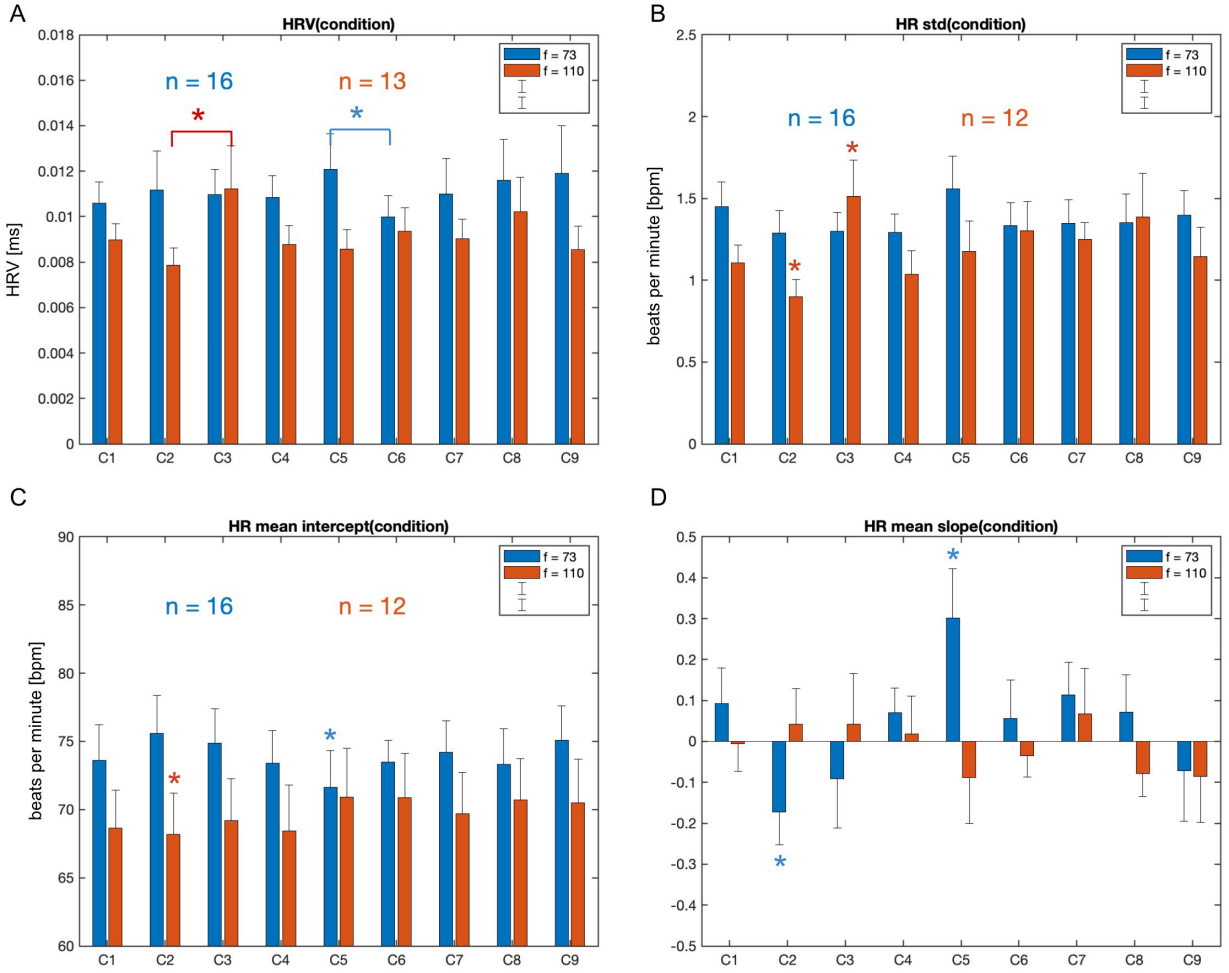
**(A–D)** Mean and standard error of HR and HRV for the 73 Hz stimulus (*n* = 16) and the 110 Hz stimulus (*n* = 13) sorted by condition (C1–C9). Conditions with significant differences (*p* < 0.05) are indicated with *. **(A)** HRV shows a significant decrease in C6 compared to C5 for the 73 Hz stimulus; and a significant increase in C3 compared to C2 for the 110 Hz stimulus. **(B)** HR standard deviation (std) shows a significant decrease in C2 compared to C1, C3, C5, C6, and C7; and a significant increase in C3 compared to C4 and C9 for the 110 Hz stimulus. **(C)** HR mean intercept shows a significant decrease in C5 compared to C2, C3, and C6 for the 73 Hz stimulus; and a significant decrease in C2 compared to C5 and C8 for the 110 Hz stimulus. **(D)** HR mean slope shows a significant decrease in C2 compared to C1, C4, C5, C7, and C8; and, a significant increase in C5 compared to C3, C6, and C9 for the 73 Hz stimulus.

Mean and standard error of HR and HRV sorted by frequency and shape of the stimulus are shown in Figures 13A–D. No significant differences were found in HRV depending on the room size of the stimulus.

Significant differences in HR standard deviation between conditions were positively correlated to the standard deviation of phase distortion of sound waves, i.e., the standard deviation of the average phase shift across all measured frequencies. The correlation was found to be significant after Bonferroni correction (*R* = 0.93, *p* < 0.00239). The correlated means of HR standard deviation and phase distortion are shown in Table 3.

**Table 3:** Significant differences in heart rate standard deviation (HR std) between conditions for the 110 Hz stimulus (as shown in Figure 13B); and corresponding differences in the standard deviation of phase distortion (ψ std) of sound waves measured at all microphones (Mics 1–6; see Figures 2B,C).

110 Hz condition	HR std (bpm)	ψ std (°)
C2—C1	−0.31	0.96
C2—C3	−0.73	−13.13
C2—C5	−0.40	0.68
C2—C6	−0.55	−0.75
C2—C7	−0.50	1.00
C3—C4	0.45	12.70
C3—C9	0.36	15.56

HR std is indicated as beats per minute (BPM). ψ std is indicated as ±degrees (−180–180°).

Significant differences in HR mean slope between conditions were positively correlated to the variability of amplitude deviation at the fundamental frequency of the sound stimulus (*R* = 0.93, *p* < 0.00081), as well as to the variability of phase shift at the fundamental frequency of the sound stimulus (*R* = 0.96, *p* < 0.00016). We also found a negative correlation between significant differences in HR mean slope and the mean phase shift of the fundamental frequency of the sound stimulus (*R* = −0.97, *p* < 0.00007). These correlations were all found to be significant after Bonferroni correction. The correlated means of HR mean slope, amplitude deviation and phase shift are shown in Table 4.

**Table 4:** Significant differences in heart rate (HR) mean slope between conditions for the 73 Hz stimulus (as shown in Figure 13D); and corresponding differences in variability of amplitude deviation (*A*_*D*_), mean phase shift (ψ) and variability of phase shift at the fundamental frequency of the sound stimulus, as measured at all microphones (Mics 1–6; see Figures 2B,C).

73 Hz condition	HR mean slope	*A_*D*_* variability (dB)	Mean ψ (°)	ψ variability (°)
C2—C1	−0.26	9.95 × 10^−3^	5.67	−3.09 × 10^−2^
C2—C4	−0.24	4.21 × 10^−3^	12.92	9.36 × 10^−2^
C2—C5	−0.45	−4.75 × 10^−2^	23.57	−0.34
C2—C7	−0.28	1.12 × 10^−2^	8.67	−4.73 × 10^−2^
C2—C8	−0.24	1.27 × 10^−3^	6.58	−2.26 × 10^−2^
C5—C3	0.37	5.99 × 10^−2^	−15.74	0.39
C5—C6	0.24	5.72 × 10^−2^	−13.49	0.40
C5—C9	0.35	5.91 × 10^−2^	−14.48	0.45

*A*_*D*_values are indicated as ±dB. ψ values are indicated as ±degrees (−180–180°).

### 3.4 EMG

**Figure 14:**
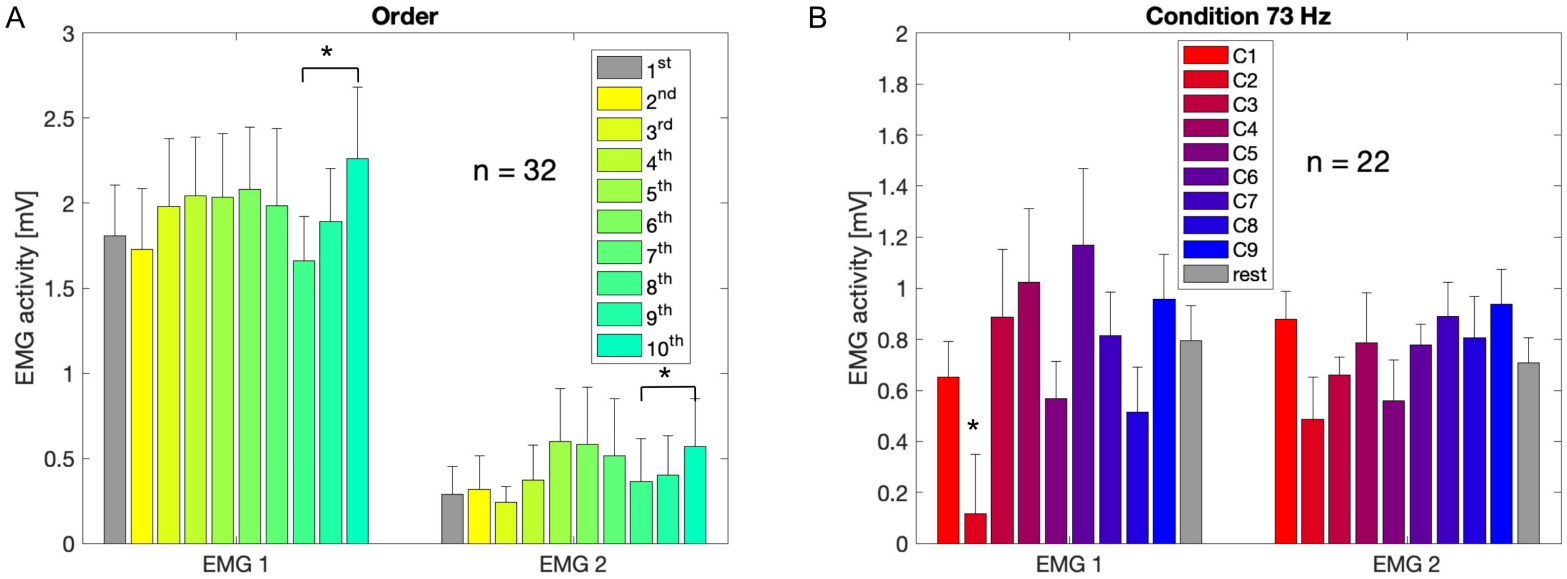
**(A, B)** Mean and standard error of EMG activity. Stimuli with significant differences between order and conditions (*p* < 0.05) are indicated with *. **(A)** EMG activity for the 73 Hz and 110 Hz stimuli combined (*n* = 32) sorted by stimulus order. The first condition is resting state (baseline). EMG channels show a significant increase from the 8th to the 10th stimulus, regardless of stimulus type and condition. **(B)** EMG activity for the 73 Hz stimulus (*n* = 22) sorted by condition (C1–C9). EMG 1 shows a significant decrease in C2 compared to C1, C3, C4, C6, C7, C8, C9, and baseline.

Mean and standard error of EMG activity sorted by stimulus order, i.e., the duration of the stimulus blocks regardless of the frequency and condition, are shown in Figure 14A. Mean and standard error of EMG activity sorted by frequency and shape of the stimulus are shown in Figure 14B. No significant differences were found in EMG activity depending on the room size of the stimulus.

Significant differences in EMG activity between conditions were negatively correlated to the phase shift of the fundamental frequency of the sound stimulus. The correlation was found to be marginally significant after Bonferroni correction (*R* = 0.78, *p* < 0.02242). The correlated means of EMG activity and phase shift are shown in Table 5.

**Table 5:** Significant differences in electromyography (EMG) activity between conditions for the 73 Hz stimulus (as shown in Figure 14B); and corresponding differences in variability of mean phase shift (ψ) at the fundamental frequency of the sound stimulus, as measured at all microphones (Mics 1–6; see Figures 2B, C).

73 Hz condition	EMG activity (mV)	Mean ψ (°)
C2—C1	−0.54	5.67
C2—C3	−0.77	7.83
C2—C4	−0.92	12.92
C2—C6	−1.08	10.08
C2—C7	−0.71	8.67
C2—C8	−0.45	6.58
C2—C9	−0.87	9.09
C2—base	−0.68	8.58

EMG is indicated in ±millivolts (mV). ψ is indicated as ±degrees (−180–180°).

### 3.5 GSR

**Figure 15:**
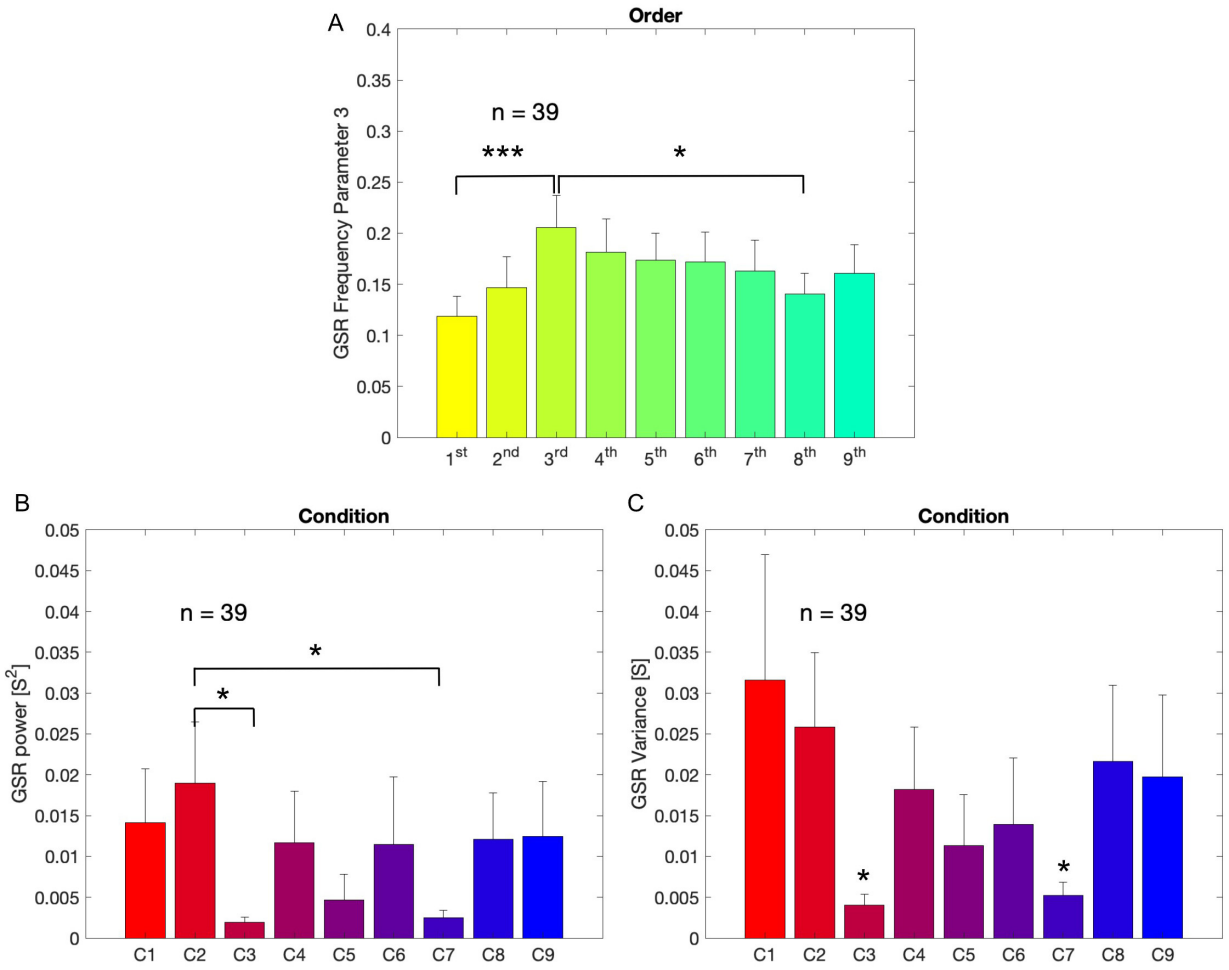
**(A–C)** Mean and standard error of GSR for the 73 Hz and 110 Hz stimuli combined (*n* = 39). Stimuli with significant differences between order and conditions (*p* < 0.05) are indicated with *. **(A)** GSR center frequency (frequency parameter 3) sorted by stimulus order. GSR center frequency shows a highly significant increase from the 1st to the 3rd stimulus (*p* < 0.0005) indicated with ***; and a significant decrease from the 3rd to the 8th stimulus, regardless of stimulus type and condition. **(B)** GSR power sorted by condition (C1–C9). GSR power shows a significant increase in C2 compared to C3 and C7. **(C)** GSR variance sorted by condition (C1–C9). GSR variance shows a significant decrease in C3 compared to C1, C2, C4, C8, and C9; and a significant decrease in C7 compared to C1, C2, C4, and C8, regardless of stimulus type.

Mean and standard error of GSR sorted by stimulus order, i.e., the duration of the stimulus blocks regardless of the frequency and condition, are shown in Figure 15A. Mean and standard error of GSR sorted by the shape of the stimulus are shown in Figures 15B,C. No significant differences were found in GSR depending on the frequency and the room size of the stimulus.

Significant differences in GSR variance between conditions were positively correlated to the variability of phase distortion of sound waves, i.e., variability of the average phase shift across all measured frequencies. The correlation was found to be marginally significant after Bonferroni correction (*R* = 0.75, *p* < 0.01994). The correlated means of GSR variance and phase distortion are shown in Table 6.

**Table 6:** Significant differences in the variance of galvanic skin response (GSR) between conditions (as shown in Figure 15C); and corresponding differences in variability of phase distortion (ψ) of sound waves measured at all microphones (Mics 1–6; see Figures 2B, C).

Condition	GSR variance	ψ variability (°)
C3—C1	−0.029	−0.51
C3—C2	−0.023	3.06 × 10^−2^
C3—C4	−0.015	−1.33
C3—C8	−0.018	6.83 × 10^−2^
C3—C9	−0.016	8.79 × 10^−2^
C7—C1	−0.027	−0.61
C7—C2	−0.021	−7.79 × 10^−2^
C7—C4	−0.013	−0.12
C7—C8	−0.016	−4.03 × 10^−2^

ψ variability is indicated as ± degrees (−180–180°).

## 4 Discussion

### 4.1 Effects of low-frequency sound on psycho-emotional state

Overall, exposure to low-frequency singing bowl sounds resulted in a significant decrease in negative emotions and a significant increase in positive emotions across all conditions. This confirms the findings of prior studies that observed the positive effects of singing bowl sounds on the emotional wellbeing of subjects (Goldsby et al., 2016; Kim and Choi, 2023; Rio-Alamos et al., 2023; Trivedi et al., 2019; Walter and Hinterberger, 2022). Although similar outcomes were observed for each of the stimulus frequencies, the 40 Hz stimulus showed the most highly significant effects across the most number of items, confirming its effectiveness for inducing emotional wellbeing (Jirakittayakorn and Wongsawat, 2017; Shakya et al., 2026).

While the singing bowl sounds induced a significant shift toward positive valence, arousal levels were balanced. Subjects reported feeling highly activated, while also feeling rested, deeply relaxed and calm after exposure to the 40 Hz stimulus, and wider awake while also feeling relaxed and calm after exposure to the 73 Hz stimulus. Outcomes for both frequencies indicate a state of restful alertness, similar to outcomes reported in studies on the effects of integrative body-mind training (Tang, 2011) and transcendental meditation (Alexander et al., 1989; Rosaen and Benn, 2006). In response to the 110 Hz stimulus, subjects reported feeling highly activated while also feeling more sleepy. We suggest that feeling highly activated was associated with a trend of increased valence, e.g., feeling greater, more superb and more happy, while simultaneously feeling more sleepy was associated with a trend of decreased arousal, e.g., feeling less nervous, anxious and tense. This is contrary to the finding that singing bowl exposure significantly decreased sleepiness (Bergmann et al., 2020). We therefore suggest that the frequency of 110 Hz may specifically induce sleepiness in subjects, which could be further investigated in future studies.

### 4.2 Combined effects of sound frequency and spatial conditions

Contrary to other studies (e.g., Hori et al., 2005; Jirakittayakorn and Wongsawat, 2017; Nogier, 2014) we found no discrete effects related to the stimulus type or frequency. Significant differences in the physiological expression of subjects were consistently dependent on a specific combination of stimulus frequency and spatial condition, i.e., the resonance of a sound in a room with a particular size and shape. While some of our results confirmed EEG patterns observed in previous studies, e.g., a significant increase in theta activity in the 40 Hz stimulus and a significant increase of high-alpha and low-beta activity in the 110 Hz stimulus (e.g., see Ahn et al., 2019; Kim and Choi, 2023) along with a significant decrease in GSR (Bidin et al., 2016), these effects did not sustain across different spatial conditions.

Findings in previous studies may have been incorrectly attributed as a discrete effect of singing bowl sounds, whereas our evaluation according to the physioacoustic model highlights the interdependency of such findings with the spatial conditions of the intervention. This also explains contrary findings in EEG (e.g., cf. Kim and Choi, 2023; Sundharakumar et al., 2021; Walter and Hinterberger, 2022) and HRV (e.g., cf. Koelsch et al., 2016; Trivedi et al., 2019; Veternik et al., 2018) across various studies investigating singing bowl sounds. We suggest that these discrepancies may depend on differences in the stimulus frequencies in combination with differences in spatial conditions, i.e., the acoustic properties of the room and the delivery methods that were used during the interventions.

Correlation with acoustic measurements reveals that significant differences in localized EEG power were strongly mediated by localized amplitude deviation of sound waves, which result from the involuntary interaction of the spatial sound stimulus and the subject in the acoustic environment. Hearing-evoked cortical response to variation in sound intensity has been observed in various studies (e.g., Di et al., 2018; Ott et al., 2013). In this study, it was demonstrated for the first time that this dependency is localizable, i.e., the areas of increase and decrease in sound pressure in the room have a spatial correspondence to areas with significant power differences in the brain. Furthermore, we find that this relationship is also phase-dependent, i.e., the frequency of variation in sound intensity shows a complex nonlinear correspondence to the frequency of significant power differences in the brain.

These findings provide new insights with implications for the emerging field of acoustic neurostimulation (Kanzler et al., 2023; Kim et al., 2023). Whereas, current approaches focus primarily on the activation and suppression of specific frequencies, spatialization of stimuli shows potential to target areas of the brain more specifically and refine targeting strategies through frequency coupling and phase-dependent modulation. Measuring and controlling for complex subject-dependent interactions in the acoustic field can inform the development of brain-responsive interfaces that modulate neural oscillations implicated in neurological and mental health disorders (Henao et al., 2020; Ortega-Robles et al., 2025) and advance diagnostics of neurological and psychiatric disorders (Longo et al., 2026).

Significant differences in autonomic balance were strongly mediated by the level of phase distortion of the sound stimulus in the acoustic environment. A negative phase shift of the fundamental frequency of the sound stimulus corresponded to a significantly increasing tendency of HR and EMG, while a positive phase shift corresponded to a decreasing tendency. This relationality was further confirmed by the correlation of the standard deviation of phase distortion and HR. The tendency of HR to gradually increase indicates activation by the sympathetic nerves and is associated with higher arousal, while increase of the standard deviation of HR indicates dominance of parasympathetic activation and lower arousal (Levy et al., 1993; Shaffer et al., 2014). In conjunction, muscle tension was also identified as an indicator of arousal (Hoehn-Saric et al., 1997). We suggest that subjects experienced involuntary shifts in arousal in response to a directional change in phase shift of the sound waves, which was caused by the complex interaction of the stimulus frequency, spatial condition and the presence of the subject in the acoustic environment.

Spatial modulation of the phase shift in low-frequency sound shows potential as a regulator of autonomic balance in subjects, which could support methods of auditory entrainment. While auditory entrainment has been effective in modulating cortical activity and induced only minimal changes in ANS parameters (Scala et al., 2025), alignment of physiological oscillatory phases at stimulus onsets showed potential in optimizing sensorimotor responses in subjects, suggesting a potential mechanism of embodied predictive processing (Muñoz-Caracuel et al., 2024).

The variability of phase distortion corresponded to arousal levels in subjects, expressed by significant differences in HR slope and GSR variance. The variability of amplitude deviations was also found corresponding to HR slope. Increase in variability of sound wave deviations may have been caused by the increase of involuntary head and body movements of the subjects during trials, which are considered significant indicators of higher physiological and emotional arousal and are more frequently triggered by increased adrenaline, stress, anxiety, or high emotional states (Mahoney and Chapman, 2004). It was outside the scope of this study to determine the causal relationship between deviations in the sound field and corresponding physiological and behavioral changes in subjects, which should be further investigated in future studies.

### 4.3 Effects of spatial conditions

Discrete effects of the spatial condition, i.e., effects of the room shape regardless of stimulus type and frequency, were observed in arousal levels of subjects as monitored by the GSR. In particular, a room in the shape of a cube and cuboctahedron induced a significant decrease in GSR power and GSR variance compared to other shapes. Prior studies reported that auditory effects of room size were primarily expressed by the level of arousal in subjects (Tajadura-Jiménez et al., 2010) and that different geometrical shapes of a room can evoke different levels of engagement and arousal (Elbaiuomy et al., 2019). It may be the subject of a future study to compare the effects of sound stimuli projected in cuboid spaces (cube and cuboctahedron associated with lower arousal) to deltoid spaces (pyramid and tetrahedron which showed a tendency to evoke higher arousal) to verify the dependency of arousal and room shape as observed in this study.

Regulating arousal in subjects by altering the virtual room shape provides a rationale for the application of spatial sound technologies in sound interventions. Few studies have investigated the neurophysiological effects of sound spatialization using binaural headphones (Heine et al., 2021) or multichannel loudspeaker configurations (Kobayashi et al., 2015; Langiulli et al., 2023). These studies have focused primarily on positioning sounds in a three-dimensional space around a subject and suggest its beneficial role in auditory processing (Heine et al., 2021) and activating the sympathetic nervous system associated with an enhanced sense of presence and immersion (Kobayashi et al., 2015). Based on the findings of this study, we suggest that room acoustic simulation according to shape-specific features can be used to target directional regulation of autonomic balance. As such, sound spatialization shows potential to improve clinical outcomes, reduce stress and aid cognitive rehabilitation in therapeutic settings.

### 4.4 Effects of stimulus duration

Discrete effects of stimulus duration were observed in arousal levels of the subjects as monitored by GSR and EMG, regardless of the frequency and spatial conditions. Arousal was significantly increased from the 1st to the 3rd stimulus, followed by a significant decrease from the 3rd until the 8th stimulus. A significant decrease in muscle activity was observed in the 8th stimulus, followed by a significant increase in activity until the 10th stimulus. As each stimulus block had a duration of 5 min, we observe that subjects were generally relaxed and low in arousal after 5 min of resting state in darkness without sound (baseline). Subjects then experienced a strong increase in arousal until 15 min of exposure to the sound stimuli. Arousal then gradually decreased again until 40 min into the experimental procedure, and increased again during the last 5–10 min, which may indicate restlessness or growing physical discomfort of sitting still after this time period.

Prior studies have reported states of deep relaxation after exposure to a 20 min intervention with singing bowls (Trivedi et al., 2019), as well as lower subjective sleepiness (Bergmann et al., 2020; Walter and Hinterberger, 2022). Stimulus durations of 30 min up to 1 h were reported to induce a lower level of arousals (Bidin et al., 2016; Goldsby et al., 2016; Walter and Hinterberger, 2022). In these studies, subjects were typically engaged with singing bowls in a lying position. In support of our findings, it was reported that during a 40 min singing bowl sound intervention with subjects in a seated position, the overall stress level first increases and begins to reduce after about 15 min and continues to follow a downward trend (Panchal et al., 2020). The observed pattern of arousal indicates that a sound intervention with the aim to increase alertness of the subject could be most effective for a duration of 15 min, while a sound intervention of 40 min is more effective for establishing deep relaxation and emotional balance. Further studies are warranted that verify the effects of stimulus duration across variable conditions.

### 4.5 Study limitations

The present study has several limitations that warrant attention. No demographic and anthropometric data on the subjects were collected other than their sex. Other individual aspects such as personality, musical background, music preference or handedness of the subjects were not regarded as variables with a major influence. Instead, individual variations between subjects were regarded as interindividual noise. However, more recent studies have specifically highlighted the confounding effects of music listening history and personal preference on psychophysiological outcomes (e.g., Lin et al., 2025; Lv et al., 2024) and the correlation between anthropometric features and acoustic deviations (Oomen et al., 2026) Future studies should elaborate on how the individual traits of subjects characterize outcomes within the physioacoustic model.

Although all subjects included in the study claimed to be proficient in English, a possible limitation could have arisen in individual responses to questionnaires due to English not being the native language. Subjects were enrolled in one of three groups and examination of each group was executed sequentially over a total time period of 12 months, which poses a risk of bias due to seasonal effects that may have influenced outcomes in each group differently. The study had a high attrition rate across some of the groups, mostly due to technical and operational failure. As a result, sample sizes were not balanced between groups and vary across outcome measures.

Due to constraints in the data processing, correlation analysis of acoustic and neurophysiological measures was performed selectively on comparisons with significant outcomes only, and for a limited amount of repeated tests for each significant outcome variable, which limits the scope of conclusions that can be drawn from the presented model. Future studies are warranted that elaborate on the model by examining the datasets for structural similarity, causal relationships, information sharing and non-linear dependencies, thus developing the model to its full predictive capacity.

In this study we assume high level functioning of the auditory system of subjects to process sensitive information in the spatial environment, such as detecting aspects pertaining to the size and shape of a room, which goes beyond more basic and familiar functions of hearing such as speech recognition or listening to music. At present it is not clear how advanced spatial processing features are precisely expressed in the brain. Future studies are warranted that examine the basic principles of higher level auditory cognition. Further research is also required to update methodologies and introduce protocols that preempt the subject's ability to engage higher level functions of cognition and response to complex sound stimuli. The reporting and discussion of results is thus limited by a lack of prior research on the subject matter.

Finally, it should be noted that the spatial conditions of sound interventions are generally poorly documented, and details on the acoustic characteristics of the test environment of sound interventions are typically not reported, which limits comparison of the outcomes of this study to those of other studies. Future studies are warranted comparing sound equipment and environments to determine standards and guidelines for sound and music-based interventions and ensure consistency across studies.

These limitations are crucial for contextualizing the discussion of our findings and should guide future research efforts that aim at further validation and verification of sound and music-based interventions and its mechanisms of action.

## Data Availability

The datasets presented in this study can be found in online repositories. The names of the repository/repositories and accession number(s) can be found below: https://osf.io/5gwjk, https://osf.io/8k9p6, https://osf.io/rh7qt.
